# Supplementary material for: A New Manganese Superoxide Dismutase Mimetic Improves Oxaliplatin-Induced Neuropathy and Global Tolerance in Mice
Source: Int J Mol Sci. 2022 Oct 26;23(21):12938. doi: 10.3390/ijms232112938 (PMC9658974; doi:10.3390/ijms232112938)
Supplement: Supplementary file 1 [file ijms-23-12938-s001.zip › ijms-1980355-supplementary.pdf]

Supporting Information

A New Manganese Superoxide Dismutase Mimetic Improves Oxaliplatin Induced Neuropathy and Global Tolerance on Mice

Caroline Prieux-Klotz [1,2,3,*,†], Henri Chédotal [3,†], Martha Zoumpoulaki [3], Sandrine Chouzenoux [1], Charlotte Chêne [1], Alvaro Lopez-Sanchez [3], Marine Thomas [1], Priya Ranjan Sahoo [3], Clotilde Policar [3], Frédéric Batteux [1], Hélène C. Bertrand [3,*], Carole Nicco [1,‡] and Romain Coriat [1,4,‡]

[1] Institut Cochin, INSERM U 1016 CNRS UMR 8104, Université de Paris, 75005 Paris, France
[2] Percy Military Hospital, Gastroenterology, 101 Avenue Henri Barbusse, 92140 Clamart, France
[3] Laboratoire des Biomolécules, LBM, Département de Chimie, Ecole Normale Supérieure, PSL University, Sorbonne Université, CNRS, 75005 Paris, France
[4] Gastroenterology, Cochin Hospital AP-HP, Université de Paris, 75014 Paris, France
* Correspondence: caroline.klotz@intradef.gouv.fr (C.P.-K.); helene.bertrand@ens.psl.eu (H.C.B.); Tel.: +33-6-7207-8783 (C.P.-K.); +33-1-4432-2440 (H.C.B.)
† These authors contributed equally to this work.
‡ These authors contributed equally to this work.

1. Experimental part.

<u>- Chemical synthesis</u>

General:

Reagents were obtained from commercial suppliers and used as received. Dry solvents were obtained from Sigma-Aldrich. ^{1}H and ^{13}C NMR spectra were recorded on a Bruker Avance 300 using solvent residuals as internal references. The following abbreviations are used: singlet (s), broad singlet (bs), doublet (d), doubled doublet (dd), triplet (t), quadruplet (q), doubled triplet (dt), and multiplet (m). High resolution mass spectra (HRMS) and mass spectra (MS) were obtained on a mass spectrometer equipped with an orbitrap mass analyzer and an electrospray ionization (ESI) ion source at the mass spectrometry platform of Sorbonne University. Reactions were followed by analytical HPLC on an Agilent Technologies 1200 series using a Higgins Proto 200 C18 (3 µm, 100 x 4.6 mm) column, and H_2O/ACN both with 0.1% TFA as mobile phase at 1 mL/min. Purifications were done by Preparative HPLC on an Agilent 1260 Infinity using a NUCLEODUR C18 (5 µm, 125 x 21 mm) column, and H_2O/ACN both with 0.1% TFA as mobile phase at 14 mL/min. UV−visible spectra were recorded on a CARY 300 Bio UV−visible spectrophotometer using a double-beam mode with media as the reference. Quartz UV-cuvettes (1.5 mL) were purchased from Hellma (12.5 x 12.5 x 45 mm).

Pt(DACH)I$_2$: K_2PtCl_4 (1.00 g, 2.41 mmol, 1.0 eq.) was dissolved in filtered H_2O (10 mL) and stirred under Ar, protected from light, for 20 min. A solution of KI (850 mg, 5.12 mmol, 2.12 eq.) in H_2O (1.6 mL) previously stirred for 20 min was added, and the resulting mixture was further stirred for 20 min. Trans-1,2-diaminocyclohexane (287 mg, 2.51 mmol, 1.04 eq.) in H_2O (700 µL) was finally added and the resulting solution was stirred at room temperature, protected from light, for 18 h. The resulting solid was filtered, washed with H_2O and dried under vacuum to afford Pt(DACH)I$_2$ as a pale yellow solid (1.214 g, 89% chemical yield).

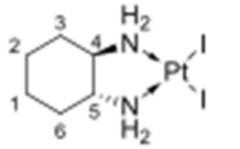

^{1}H NMR (DMSO-d$_6$, 300 MHz) *δ* 6.65 – 5.42 (m, 4H, 2 x NH_2), 2.34 (bs, 2H, H_4+H_5), 1.97 (dd, 2H, H_3+H_6), 1.51 (bs, 2H, H_1+H_2), 1.29 (bs, 2H, $H_{3'}$+$H_{6'}$), 1.02 (m, 2H, $H_{1'}$+$H_{2'}$); **^{13}C NMR (DMSO-d$_6$, 75 MHz)** *δ* 63.7 (CH, C_4/C_5), 60.3 (CH, C_4/C_5), 31.8 (CH_2, C_3/C_6), 31.2 (CH_2, C_3/C_6), 24.0 (CH_2, C_1/C_2), 23.8 (CH_2, C_1/C_2). *Spectral data in accordance with those previously reported.*[1]

Pt(DACH)ox: Oxalic acid (323.0 mg, 3.59 mmol, 1.0 eq.) was added in a falcon and suspended in H_2O (40 mL). Then NaOH (287 mg, 7.18 mmol, 2.0 eq.) was added, followed by silver nitrate (1.220 g, 7.18 mol, 2.0 eq.) and the solution was stirred at room temperature protected from light for 15 min. The precipitate was separated by centrifugation (6 min, 7500 rpm), washed with cold H_2O, and dried under vacuum to afford silver oxalate as a white powder (1.078 g, 99% chemical yield). Then, Pt(DACH)I_2 (1.214 g, 2.16 mmol, 1.0 eq.) and silver oxalate (655 mg, 2.16 mmol, 1.0 eq.) were suspended in water (80 mL) and the mixture was stirred under Ar, protected from light at room temperature for 12 days. The solid formed was separated by centrifugation (6 min, 7500 rpm) and the solution was lyophilized to afford Pt(DACH)ox as a pale yellow solid (723 mg, 84% chemical yield).

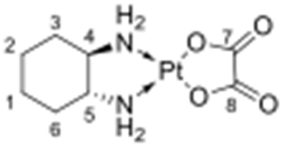

^{1}H NMR (DMSO-d$_6$, 300 MHz) *δ* 6.10 (d, *J* = 8.4 Hz, 2H, NH_2), 5.51 - 5.19 (m, 2H, NH_2), 2.07 – 1.91 (m, 2H, H_4+H_5), 1.82 (d, *J* = 12.3 Hz, 2H, H_3+H_6), 1.45 (d, *J* = 8.1 Hz, 2H, H_1+H_2), 1.31 – 1.11 (m, 2H, $H_{3'}$+$H_{6'}$), 1.10 – 0.90 (m, 2H, $H_{1'}$+$H_{2'}$); **^{13}C NMR (DMSO-d$_6$, 75 MHz)** *δ* 165.9 (2 x Cq, C_7+C_8), 61.8 (2 x CH, C_4+C_5), 31.5 (2 x CH_2, C_3+C_6), 24.1 (2 x CH_2, C_1+C_2); **HRMS** calculated for $C_8H_{14}N_2O_4PtNa$ (M+Na$^+$): 420.0494, found 420.0498. *Spectral data in accordance with those previously reported.*[2]

Pt(DACH)ox(OH)$_2$: Pt(DACH)ox (326 mg, 0.821 mmol, 1.0 eq.) was suspended in H_2O (30 mL) and H_2O_2 (30% w/v, 1.64 mL, 18 eq.) was added. The solution was stirred at room temperature, protected from light, for 2 days. The mixture was diluted with acetone (10 mL) and kept in the fridge at 4 °C for 24 h. The white solid formed was filtered, washed with acetone and dried under vacuum to afford Pt(DACH)ox(OH)$_2$ as a white solid (259 mg, 73% chemical yield).

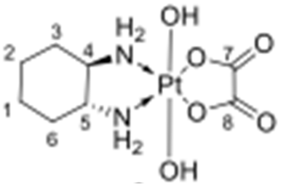

^{1}H NMR (D_2O, 300 MHz) *δ* 2.96 – 2.79 (m, 2H, H_4+H_5), 2.31 (d, *J* = 12.3 Hz, 2H, H_3+H_6), 1.79 – 1.48 (m, 4H, H_1+H_2+$H_{3'}$+$H_{6'}$), 1.39 - 1.18 (m, 2H, $H_{1'}$+$H_{2'}$); **^{13}C NMR (D_2O, 75 MHz)** *δ* 61.5 (2 x CH, C_4+C_5), 30.9 (2 x CH_2, C_3+C_6), 23.5 (2 x CH_2, C_1+C_2); **HRMS** calculated for $C_8H_{17}N_2O_6Pt$ (M+H$^+$): 432.0729, found 432.0727.

Pt(DACH)ox(OH)(OAc): A solution of Pt(DACH)ox (359 mg, 0.904 mmol, 1.0 eq.) and H_2O_2 (30% w/v, 307 µL, 2.708 mmol, 3.0 eq.) in glacial acetic acid (18 mL) was stirred at room temperature, under Ar, protected from light, for 2 days. The mixture was filtered and evaporated. The residue was taken up in EtOAc, triturated and the solvent was discarded. The solid was dried under vacuum to afford Pt(DACH)ox(OH)(OAc) as a white solid (304 mg, 71% chemical yield).

---

[1] Cirri D., Pillozzi S., Gabbiani C., Tricomi J., Bartoli G., Stefanini M., Michelucci E., Arcangeli A., Messoria L., Marzo T., *Dalton Trans.*, **2017**, *46*, 3311-3317.

[2] Varbanov H. P., Ortiza D., Höfer D., Menina L., Galanski M., Keppler B. K., Dyson P. J., *Dalton Trans.*, **2017**, *46*, 8929-8932.

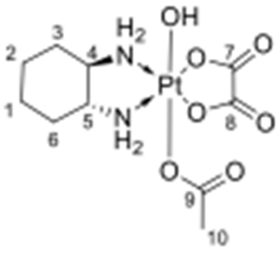

^{1}H NMR (D_2O, 300 MHz) δ 3.00 – 2.81 (m, 2H, H_4+H_5), 2.31 (d, *J* = 10.5 Hz, 2H, H_3+H_6), 2.09 (s, 3H, H_{10}), 1.81 – 1.50 (m, 4H, H_1+H_2+$H_{3'}$+$H_{6'}$), 1.34 – 1.21 (m, 2H, $H_{1'}$+$H_{2'}$); **^{13}C NMR (D_2O, 75 MHz)** δ 61.3 (2 x CH, C_4+C_5), 30.9 (2 x CH_2, C_3+C_6), 23.4 (2 x CH_2, C_1+C_2), 22.7 (CH_3, C_{10}); **HRMS** calculated for $C_{10}H_{18}N_2O_7PtNa$ ($M+Na^+$): 496.0654, found 496.0658.

1C1A: To a solution of 1.TFA[3] (726.1 mg, 1.379 mmol, 1.0 eq.) in absolute ethanol (54 mL) was added ethyl glyoxylate (50% in toluene, 0.474 mL, 2.322 mmol, 1.68 eq.) and TFA (62 µL, 0.774 mmol, 0.56 eq.). The solution was stirred at room temperature for 2 h and $NaBH_3CN$ (97.2 mg, 1.547 mmol, 1.12 eq.) was added. The resulting mixture was stirred at room temperature for 18 h. The pH was adjusted to pH 9 by addition of an 1M aqueous NaOH solution and the solution was concentrated. The mixture was diluted with DCM and H_2O, extracted thrice with DCM. The combined organic phases were washed with brine, dried over Na_2SO_4, filtered and evaporated. The residue was purified by RP-HPLC (NUCLEODUR, 10 to 30 % ACN, 30 min), injection as concentrated DMF solution. Fractions of interest were lyophilized to afford the functionalized ester (TFA salt) as a colourless oil (250.5 mg, 33% chemical yield).

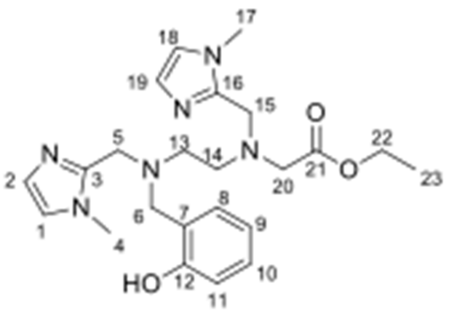

HPLC (5 to 100% ACN, 10 min): rt 4.610 (> 95%) ; **^{1}H NMR (300 MHz, MeOD)** δ 7.49 (d, *J* = 1.8 Hz, 1H, H^{Im}), 7.43 (d, *J* = 1.8 Hz, 1H, H^{Im}), 7.39 (d, *J* = 1.8 Hz, 1H, H^{Im}), 7.37 (d, *J* = 1.8 Hz, 1H, H^{Im}), 7.18 – 7.07 (m, 2H, H^{Ph}), 6.84 – 6.73 (m, 2H, H^{Ph}), 4.24 – 4.07 (m, 6H, H_5+H_{15}+H_{22}), 3.82 (s, 3H, H_{17}), 3.79 (s, 2H, H_{20}), 3.76 (s, 3H, H_4), 3.47 (s, 2H, H_6), 3.04 – 2.83 (m, 4H, H_{13}+H_{14}), 1.24 (t, *J* = 7.2 Hz, 3H, H_{23}); **^{13}C NMR (75 MHz, MeOD)** δ 172.6 (Cq, C_{21}), 157.3 (Cq, C_{12}), 146.5 (Cq, C_3), 145.8 (Cq, C_{16}), 132.8 (CH, C^{Ph}), 130.9 (CH, C^{Ph}), 125.6 (CH, C^{Im}), 125.4 (CH, C^{Im}), 123.4 (Cq, C_7), 120.9 (CH, C^{Ph}), 120.0 (CH, C^{Im}), 119.6 (CH, C^{Im}), 116.6 (CH, C^{Ph}), 62.2 (CH_2, C_{22}), 55.9 (2 x CH_2, C_6+C_{20}), 54.2 (CH_2, C_{14}), 53.2 (CH_2, C_{13}), 49.8 (CH_2, C_{15}), 49.4 (CH_2, C_5), 35.1 (CH_3, C_{17}), 35.0 (CH_3, C_4), 14.6 (CH_3, C_{23}).

To a solution of the ester (TFA salt) (250.5 mg, 0.452 mmol, 1.0 eq.) in acetone (3 mL) was added 1 M aqueous NaOH solution (4.6 mL). The solution was stirred at room temperature for 1 day and H_2O was added. The resulting mixture was extracted with DCM. The aqueous phase was acidified to pH 2 with conc. HCl and freeze-dried. The residue was purified by RP-HPLC (NUCLEODUR, 5 to 50 % ACN in 30 min) to afford 1C1A.TFA as a colourless oil (127.0 mg, 53% chemical yield).

[3] Cisnetti F, Lefèvre AS, Guillot R, Lambert F, Blain G, Anxolabéhère-Mallart E, et al. A New Pentadentate Ligand Forms Both a Di- and a Mononuclear MnII Complex: Electrochemical, Spectroscopic and Superoxide Dismutase Activity Studies. *Eur J Inorg Chem.* **2007** (28), 4472‑80

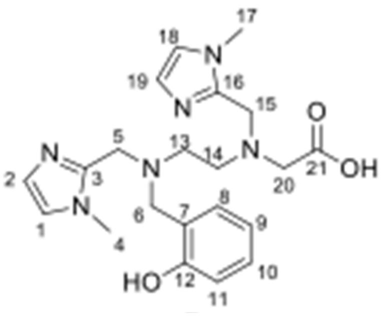

HPLC (5 to 100% ACN, 10 min): rt 3.851 (> 95%); **^{1}H NMR (300 MHz, D_2O)** δ 7.54 (d, J = 2.0 Hz, 1H, H^{Im}), 7.46 (d, J = 2.0 Hz, 1H, H^{Im}), 7.40 – 7.37 (m, 2H, H^{Im}), 7.35 – 7.29 (m, 2H, H^{Ph}), 7.02 – 6.94 (m, 1H, H^{Ph}), 6.90 – 6.85 (m, 1H, H^{Ph}), 4.88 (s, 2H, H_5), 4.46 (s, 2H, H_{20}), 4.04 (s, 2H, H_{15}), 3.84 (s, 3H, H_{17}), 3.73 (s, 3H, H_4), 3.65 – 3.53 (m, 4H, H_6+H_{14}), 3.27 (t, J = 5.7 Hz, 2H, H_{13}); **^{13}C NMR (75 MHz, D_2O)** δ 175.6 (Cq, C_{21}), 154.8 (Cq, C_{12}), 142.8 (Cq, C_3), 135.8 (Cq, C_{16}), 132.3 (CH, C^{Ph}), 131.7 (CH, C^{Ph}), 125.6 (CH, C^{Im}), 124.3 (CH, C^{Im}), 121.0 (CH, C^{Ph}), 120.7 (CH, C^{Im}), 118.5 (CH, C^{Im}), 118.3 (CH, C^{Im}), 115.89 (Cq, C_7), 115.4 (CH, C^{Ph}), 55.8 (CH_2, C_{20}), 54.8 (CH_2, C_6), 54.3 (CH_2, C_{14}), 49.7 (CH_2, C_{13}), 48.0 (CH_2, C_{15}), 46.3 (CH_2, C_5), 35.0 (CH_3, C_{17}), 34.2 (CH_3, C_4).

Pt(DACH)ox(OH)(1C1A) (OxPt-1OH-1C1A):

Pt(DACH)ox$(OH)_2$ (45.0 mg, 0.104 mmol, 1.0 eq.) was added to a solution of 1C1A.TFA (66.0 mg, 0.125 mmol, 1.2 eq.), TBTU (50.3 mg, 0.157 mmol, 1.5 eq.) and Et_3N (29.1 µL, 0.208 mmol, 2.0 eq.) in anhydrous DMF (4.6 mL) and the mixture was stirred at room temperature protected from light under Ar for 3 days. The mixture was diluted with water, freeze-dried and purified by RP-HPLC (NUCLEODUR, 5 to 50% ACN in 30 min), fractions were collected and lyophilized to afford Pt(DACH)ox(OH)(1C1A)·TFA as a white solid (86.0 mg, 41% chemical yield).

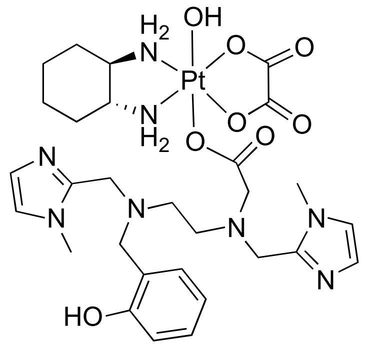

HPLC (5 to 50% ACN, 10 min): rt 5.734 (> 95%); **^{1}H-NMR (D_2O, 300 MHz)** δ 7.35-7.32 (m, 2H, CH^{Im}), 7.25-7.23 (m, 2H, CH^{Im}), 7.20-7.16 (m, 2H, CH^{Ph}), 6.90 (t, J = 7.8 Hz, 1H, CH^{Ph}), 6.80 (d, J = 7.8 Hz, 1H, CH^{Ph}), 4.09-4.04 (m, 4H, N-C<u>H2</u>-X x2), 3.82 (s, 2H, N-C<u>H2</u>-X), 3.70 (s, 6H, N-C<u>H3</u> x2), 3.49 (s, 2H, N-C<u>H2</u>-X), 2.89-2.72 (m, 6H, N-C<u>H2</u>-C<u>H2</u>-N + CH^{DACH}), 2.30-2.27 (m, 2H, $CH2^{DACH}$), 1.65-1.56 (m, 4H, $CH2^{DACH}$), 1.29-1.21 (m, 2H, $CH2^{DACH}$); **^{13}C-NMR (D_2O, 75 MHz)** δ 179.9 (C=O), 166.1 (ox), 154.5 (Ph Cq-OH), 144.1 (Cq), 142.7 (Cq), 131.2, 130.0, 123.9, 123.8, 121.5, 120.6, 119.4, 118.2, 115.5, 114.4, 61.8, 61.3, 55.0, 52.5, 51.2, 48.2, 47.7, 34.0, 33.9, 30.9, 23.4; **HRMS (ESI+)** m/z: calculated for $C_{29}H_{43}N_8O_8Pt$ $[M+H]^+$ (100%): 826.2848, found: 826.2873; calculated for $C_{31}H_{42}F_3N_8O_9Pt$ $[M-OH+CF3COOH]^+$ (94%): 922.2675, found: 922.2704.

Pt(DACH)ox$(1C1A)_2$ (OxPt-2-1C1A): Pt(DACH)ox$(OH)_2$ (51.0 mg, 0.118 mmol, 1.0 eq.) was added to a solution of $enPI_2CH_2CO_2H$·TFA (130.5 mg, 0.248 mmol, 2.1 eq.), TBTU (114.0 mg, 0.355 mmol, 3.0 eq.) and Et_3N (66 µL, 0.472 mmol, 4.0 eq.) in dry DMF (5.2 mL) and the mixture was stirred at room temperature, protected from light under Ar for 2 days. The mixture was filtered, then evaporated, retaken in H_2O, and stored in the fridge at 4°C for a week. Then it was purified by RP-HPLC (NUCLEODUR, 5 to 30% ACN in 30 min), fractions were collected and lyophilized to afford Pt(DACH)ox$(1C1A)_2$·TFA as a white solid (100.0 mg, 62% chemical yield).

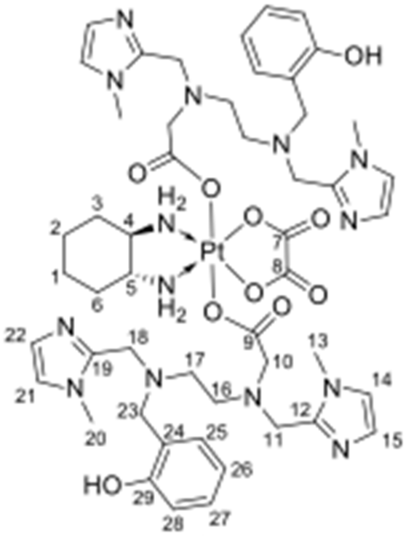

HPLC (5 to 50% ACN, 10 min): rt 6.538 (> 95%); **^{1}H NMR (300 MHz, MeOD)** *δ* 7.47 (d, *J* = 2.1 Hz, 2H, CH^{Im}), 7.42 (d, *J* = 2.1 Hz, 2H, CH^{Im}), 7.37 – 7.32 (m, 4H, CH^{Im}), 7.15 – 7.04 (m, 4H, CH^{Ph}), 6.82 – 6.70 (m, 4H, CH^{Ph}), 4.14 (s, 4H, H_{11}), 4.03 (s, 4H, H_{18}), 3.79 (s, 6H, H_{13}/H_{20}), 3.75 (s, 6H, H_{13}/H_{20}), 3.71 (s, 4H, H_{23}), 3.53 (s, 4H, H_{10}), 2.93 – 2.71 (m, 10H, $H_4+H_5+H_{16}+H_{17}$), 2.26 (d, *J* = 12.0 Hz, 2H, CH_2, H_3+H_6), 1.74 – 1.47 (m, 4H, CH_2, $H_1+H_2+H_{3'}+H_{6'}$), 1.36 – 1.19 (m, 2H, CH_2, $H_{1'}+H_{2'}$); **^{13}C NMR (75 MHz, MeOD)** *δ* 180.5 (2 x Cq, C_9), 167.7 (2 x Cq, C_{29}), 157.2 (2 x Cq, C_7+C_8), 146.5 (2 x Cq, C_{19}), 146.4 (2 x Cq, C_{12}), 132.8 (2 x CH, C^{Ph}), 130.7 (2 x CH, C^{Ph}), 125.6 (2 x CH, C^{Im}), 125.2 (2 x CH, C^{Im}), 123.7 (2 x Cq, C_{24}), 121.0 (2 x CH, C^{Ph}), 120.0 (2 x CH, C^{Im}), 119.7 (2 x CH, C^{Im}), 116.5 (2 x CH, C^{Ph}), 63.1 (2 x CH, $C_{4+}C_5$), 57.5 (2 x CH_2, C_{10}), 55.8 (2 x CH_2, C_{23}), 54.2 (2 x CH_2, C_{17}), 53.2 (2 x CH_2, C_{16}), 49.4 (2 x CH_2, C_{11}), 49.1 (2 x CH_2, C_{18}), 35.1 (2 x CH_3, C_{13}/C_{20}), 35.0 (2 x CH_3, C_{13}/C_{20}), 25.1 (2 x CH_2, C_3+C_6), 24.4 (2 x CH_2, C_1+C_2); **HRMS** calculated for $C_{50}H_{69}N_{14}O_{10}Pt$ ($M+H^+$): 1220.4964, found 1220.5003.

Pt(DACH)ox(OAc)(1C1A) (OxPt-1-1C1A): A solution of 1C1A·TFA (88.9 mg, 0.169 mmol, 1.6 eq.) and DCC (97.8 mg, 0.474 mmol, 4.4 eq.) in dry DMF (1 mL) was sonicated for 15 min and centrifuged (7 min, 7500 rpm). The supernatant was added slowly to a solution of Pt(DACH)ox(OH)(OAc) (51.0 mg, 0.108 mmol, 1.0 eq.) in dry DMF (5 mL). The mixture was stirred at room temperature, protected from light, under Ar for 2 days. The solution was evaporated, and the residue was purified by RP-HPLC (NUCLEODUR, 5 to 30% ACN, 30 min), impure fractions of interest were purified again by RP-HPLC (NUCLEODUR, 5 to 20% ACN, 30 min). Resulting fractions were lyophilized to afford Pt(DACH)ox(OAc)(1C1A).TFA salt as a white solid (18.4 mg, 17% chemical yield).

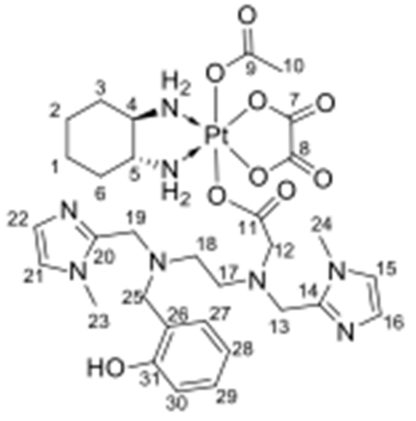

HPLC (5 to 50% ACN, 10 min): rt 5.551 (> 95%); **^{1}H NMR (300 MHz, MeOD)** *δ* 7.47 (d, *J* = 1.5 Hz, 1H, CH^{Im}), 7.42 (d, *J* = 1.5 Hz, 1H, CH^{Im}), 7.35 (s, 2H, CH^{Im}), 7.12 - 7.04 (m, 2H, CH^{Ph}), 6.83 – 6.67 (m, 2H, CH^{Ph}), 4.13 (s, 2H, H_{13}), 4.01 (s, 2H, H_{19}), 3.77 (m, 6H, $H_{23}+H_{24}$), 3.69 (s, 2H, H_{25}), 3.48 (s, 2H, H_{12}), 2.91 - 2.68 (m, *J* = 5.1 Hz, 6H, $H_4+H_5+H_{17}+H_{18}$), 2.26 (d, *J* = 12.0 Hz, 2H, H_3+H_6), 2.06 (s, 3H, H_{10}), 1.77 – 1.47 (m, 4H, $H_1+H_2+H_{3'}+H_{6'}$), 1.38 – 1.16 (m, 2H, $H_{1'}+H_{2'}$); **^{13}C NMR (75 MHz, MeOD)** *δ* 168.9 (Cq, C_9), 166.4 (Cq, C_{11}), 158.7 (Cq, C_{31}), 148.1 (2 x Cq, C_7+C_8), 132.8 (CH, C^{Ph}), 130.3 (CH, C^{Ph}), 125.6 (CH, C^{Im}), 125.2 (CH, C^{Im}), 123.8 (Cq, C_{26}), 120.9 (CH, C^{Ph}), 119.7 (2 x CH, C^{Im}), 116.5 (CH, C^{Ph}), 63.1 (2 x CH, C_4+C_5), 57.3 (CH_2, C_{12}), 55.8 (CH_2, C_{25}), 54.2 (2 x CH_2, $C_{17}+C_{18}$), 49.2 (CH_2, C_{13}), 48.8 (CH_2, C_{19}), 35.2 (2 x CH_3, $C_{23}+C_{24}$), 32.6 (2 x CH_2, C_3+C_6), 25.2 (2 x CH_2, C_1+C_2), 23.0 (CH_3, C_{10}); **HRMS** calculated for $C_{31}H_{46}N_8O_9Pt$ ($M+2H^+$): 434.6512, found 434.6514.

<u>- UV-Visible titrations:</u>

The final compounds were purified by RP-HPLC and lyophilized. Each ligand was then solubilized to make a stock solution S_0 with around 25 mg in 700 µL milliQ H_2O. Then 6 µL of this solution was diluted in 39 µL of 4-(2-hydroxyethyl)-1-piperazineethanesulfonic acid (HEPES) buffer (50 mM, pH 7.4) to afford S_1 (45 µL with C_0 = 7.5 C_1). A stock solution of $ZnCl_2$

(or $MnCl_2$) was prepared before each measure using anhydrous $ZnCl_2$ (or $MnCl_2$) salt dissolved in HEPES buffer (50 mM, pH 7.4) to afford 0.1 M solution which was further diluted to 10 mM with HEPES. The concentration of ligand stock solution was then determined by UV-visible titration with successive addition of $ZnCl_2$ (or $MnCl_2$), by following the absorbance at 288 nm as follows. The absorbance of a ligand solution (40 µL S_1 + 1460 µL HEPES (50 mM, pH 7.4)) was measured between 200 nm and 400 nm. Then, 4 µL of $ZnCl_2$ (or $MnCl_2$) solution (10 mM in HEPES 50 mM pH 7.4) were added several times, the cuvette was stirred manually, and the absorbance was recorded between 200 nm and 400 nm. Upon addition of $ZnCl_2$, the absorbance at 288 nm, corresponding to the maximum absorption wavelength of the zinc complex, increases until saturation, corresponding to a fully coordinated ligand. The absorbance at 288 nm was plotted as function of the $ZnCl_2$ volume added in the cuvette. Equivalence volume (V_{eq} $ZnCl_2$) (Figure S1) was used to determine the exact ligand concentration in the initial solution was calculated. The individual titrations are described below.

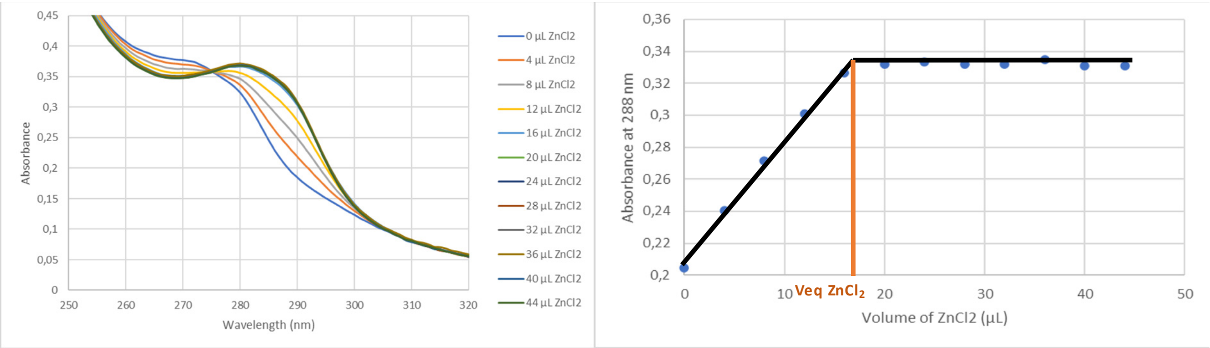

Figure S1. Example of UV-Visible titration with $ZnCl_2$.

- Stability followed by HPLC:

The stability of the uncoordinated conjugates in HEPES buffer (50 mM, pH 7.4) and the reduction rate of the Pt(IV) conjugates (uncoordinated or in the presence of Mn) by sodium ascorbate (NaAsc) was studied by analytical HPLC (method: C18 column, 5% to 100 ACN in 10 min). Samples were prepared using a stock solution of titrated platinum species at 0.1 M in H_2O. A stock solution of reductive agent was prepared by dissolving Na-Asc in HEPES (50 mM, pH 7.4), to afford 90 mM solutions. Then an Eppendorf was charged with 12 µL of platinum solution and diluted with 388 µL of either HEPES (50 mM, pH 7.4) alone or of the reductive solution to afford 400 µL at 3 mM in Pt, without or with around 30 times excess in reductive agent.

The influence of the presence of $MnCl_2$ was measured by adding 1 eq. of Mn (2 eq. for the bis-conjugate OxPt-2-1C1A) by platinum species. A stock solution was prepared by dissolving $MnCl_2$ in HEPES (50 mM, pH 7.4) to afford a 0.1 M solution. Then an Eppendorf was charged with 12 µL of platinum solution, 12 µL of Mn solution (24 µL for the bis-conjugate) and completed to 400 µL with HEPES (50 mM, pH 7.4) alone (no reductive agent) or with a 90 mM NaAsc solution in HEPES (50 mM, pH 7.4), to afford a solution at 3 mM in Pt conjugate and Mn salt (6 mM for the bis-conjugate), without or with around 30 times excess in NaAsc, in HEPES (50 mM, pH 7.4).

Reactivity rates or reduction rates were followed by injecting 40 µL of the solution on HPLC every hour and by measuring the area of the peak at 220 nm corresponding to the conjugate.

- Mass spectroscopy:

Mass spectroscopy analysis was performed at ESPCI Paris in the Spectrométrie de Masse Biologique et Protéomique (SMBP) laboratory with a QqOrbitrap in ESI+ mode with direct infusion at 10 µL/min and measured from 150 to 2000 m/z. A fresh stock solution at 100 mM in $MnCl_2$ (or $ZnCl_2$) was prepared in MilliQ H_2O, then it was diluted with MilliQ H_2O to make 100 µL of metal solution at 20 mM. A solution at 10 mM of titrated conjugate in H_2O was also prepared. Then, a solution at 5 mM in coordinated conjugate was prepared with 10 µL of the conjugate solution at 10 mM (1 eq.), 6 µL of $MnCl_2$ (or $ZnCl_2$) at 20 mM (1.2 eq), 10 µL of MQ H_2O. The samples taken for MS measurement consisted in an Eppendorf charged with 245 µL of NH_4CO_3 buffer (50 mM, pH 7.4) and 5 µL of the previous 5 mM solution to afford 250 µL at 100 µM. They were injected as 20 µM solution in 80% ACN and 20% NH_4CO_3 buffer (50 mM, pH 7.4) or MilliQ H_2O.

- Intrinsic SOD activity as for the McCord-Fridovich assay:

The SOD activity of Mn1, Mn1C1A and the 3 conjugates (OxPt-1OH-Mn1C1A, OxPt-1-Mn1C1A, OxPt-2-Mn1C1A) was determined by the McCord-Fridovich assay using the xanthine (200 µM)/xanthine oxidase system to produce superoxide, and ferricytochrome c (22µM) (see Table 1).[4-6] In this assay, superoxide is produced continuously by a xanthine/xanthine oxidase system and reduces ferricytochrome *c*, which is used as superoxide marker.

Stock solutions of ligands were prepared in milliQ H_2O at 10 mM. The 1:1 complexes were freshly prepared in HEPES buffer (50 mM, pH 7.53) at 50 µM (1 mL) by dilution of a fresh 1 mM solution in HEPES (89 µL buffer + 10 µL ligand + 1 µL of a 100 mM fresh $MnCl_2$ solution). The measurements were done in duplicate and given as a mean value.

IC_{50} is the concentration for which the kinetics of the reduction of ferricytochrome *c* is divided by two. This is also the concentration at which 50% of the superoxide produced reacted with the compound assayed. From this value, the constant k_{McCF} is determined using the following equation using the reported k_{McCF} for ferricytochrome c (2.6 x 10^5 $M^{-1}.s^{-1}$ at pH 7.8).[4-6]

$$IC_{50} \text{ x } k_{McCF} = [\text{cyt c}] \text{ x } k_{cytc}$$

2. Results.

- UV-Vis titrations

The ligand conjugates were titrated by UV-Vis spectrometry to precisely determine the concentrations of stock solutions and subsequently form the Mn complex *in situ* for further evaluations. Successive additions of a $MnCl_2$ or $ZnCl_2$ solution of known concentration in a ligand solution are performed. The maximum absorption wavelength changes during complexation of the SOD mimic ligand. Titration of the ligand 1C1A with $MnCl_2$ shows the typical curves obtained (Figure S2).

---

[4] J. M. McCord and I. Fridovich, *J. Biol. Chem.*, **1969**, 244, 6049-6055.

[5] S. Durot, C. Policar, F. Cisnetti, F. Lambert, J.-P. Renault, G. Pelosi, G. Blain, H. Korri-Youssoufi and J.-P. Mahy, *Eur. J. Inorg. Chem.*, **2005**, 3513-3523.

[6] C. Policar, in *Redox Active Therapeutics*, eds. J. S. Reboucas, I. Batinic-Haberle, I. Spasojevic, D. S. Warner and D. St. Clair, Springer, 2016, ch. Chapter 17, pp. 125-164.

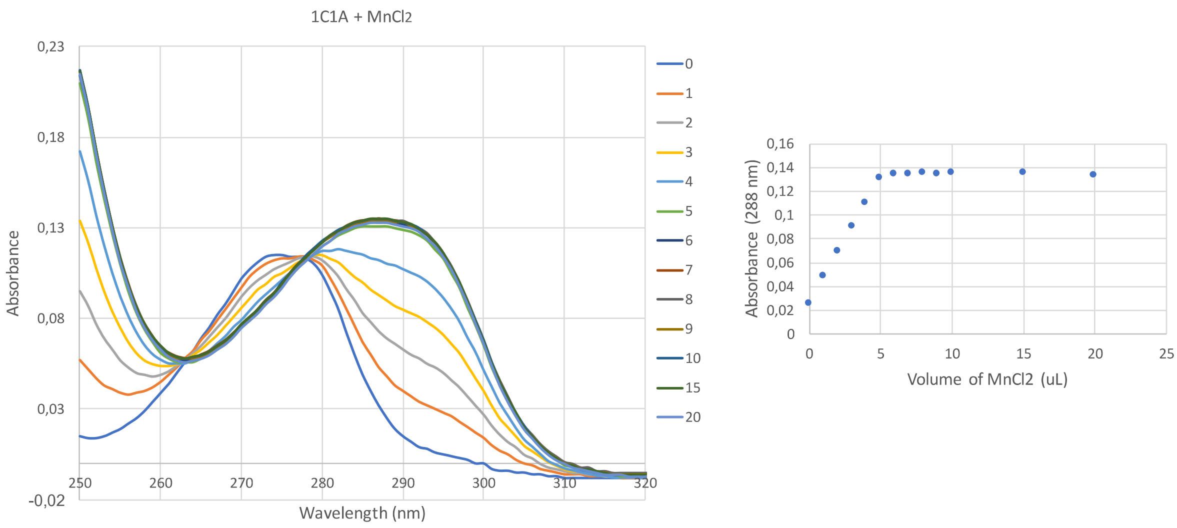

<u>Figure S2.</u> Titration curves of 1C1A with $MnCl_2$ (volume added in µL, 10 mM in HEPES 50 mM pH 7.4) and plot of the absorbance of 288 nm as a function of $MnCl_2$ volume added.

Titration with zinc known to bind with better affinity to ligand 1 but without any possible redox reactivity, was performed in first instance. Figure S3 shows a typical titration experiment of OxPt-1-1C1A by $ZnCl_2$, with a distinct change in the maximum of absorption (λ_{abs}^{max} 284 nm, attributed to phenolate to metal charge transfer). The same was observed with the bis-conjugate OxPt-2-1C1A (Figure S4).

When the conjugates were titrated with a solution of $MnCl_2$, the spectral signatures were different with no clear change in the maximum of absorbance, pointing at an additional reactivity.

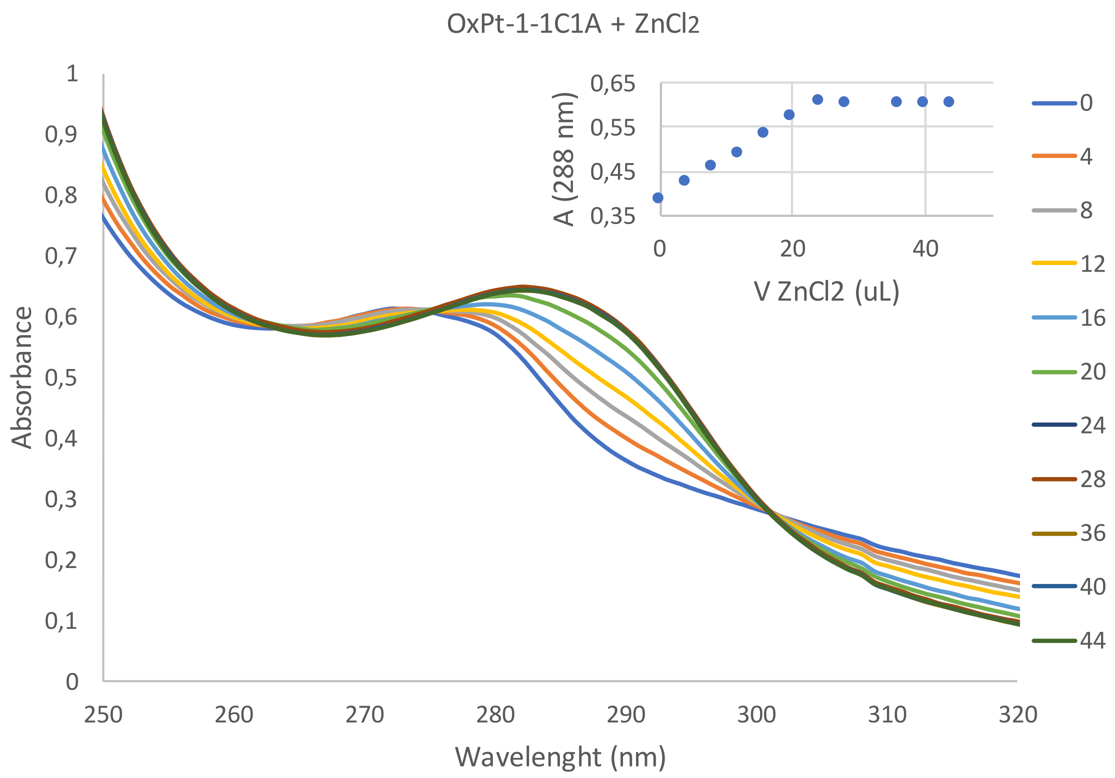

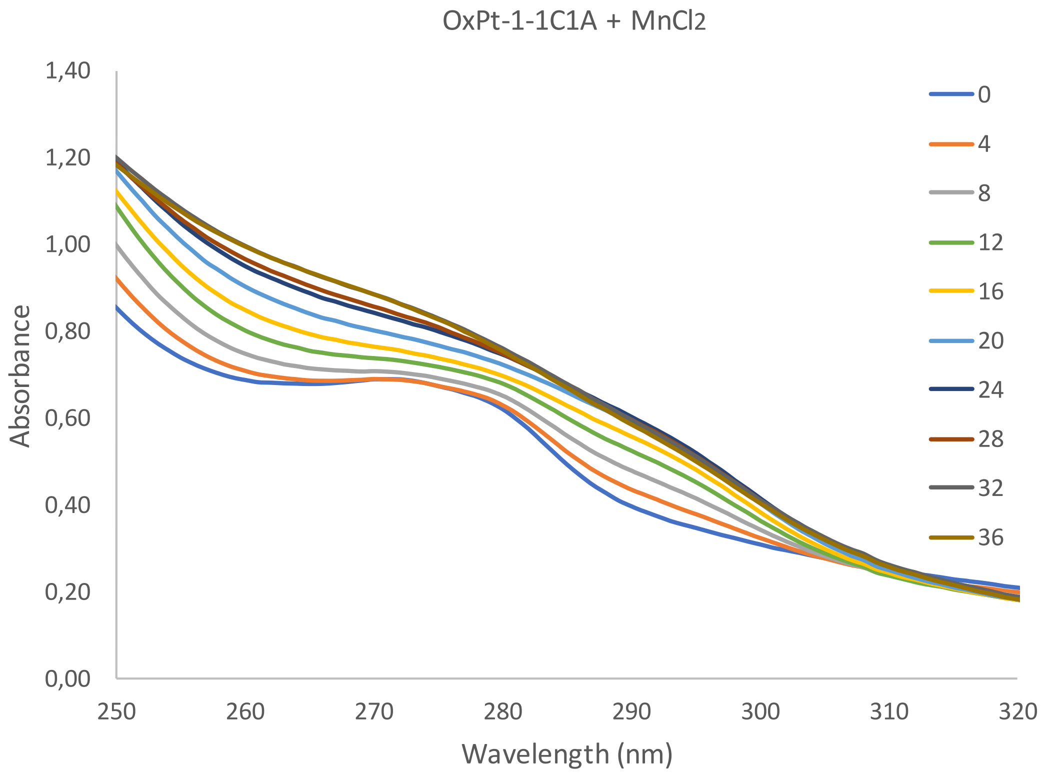

<u>Figure S3.</u> Titration curves of OxPt-1-1C1A. Top: with $ZnCl_2$ (volume added in µL, 10 mM in HEPES 50 mM pH 7.4) with plot of the absorbance of 288 nm as a function of $ZnCl_2$ volume added as insert; Bottom: with $MnCl_2$ (volume added in µL, 10 mM in HEPES 50 mM pH 7.4).

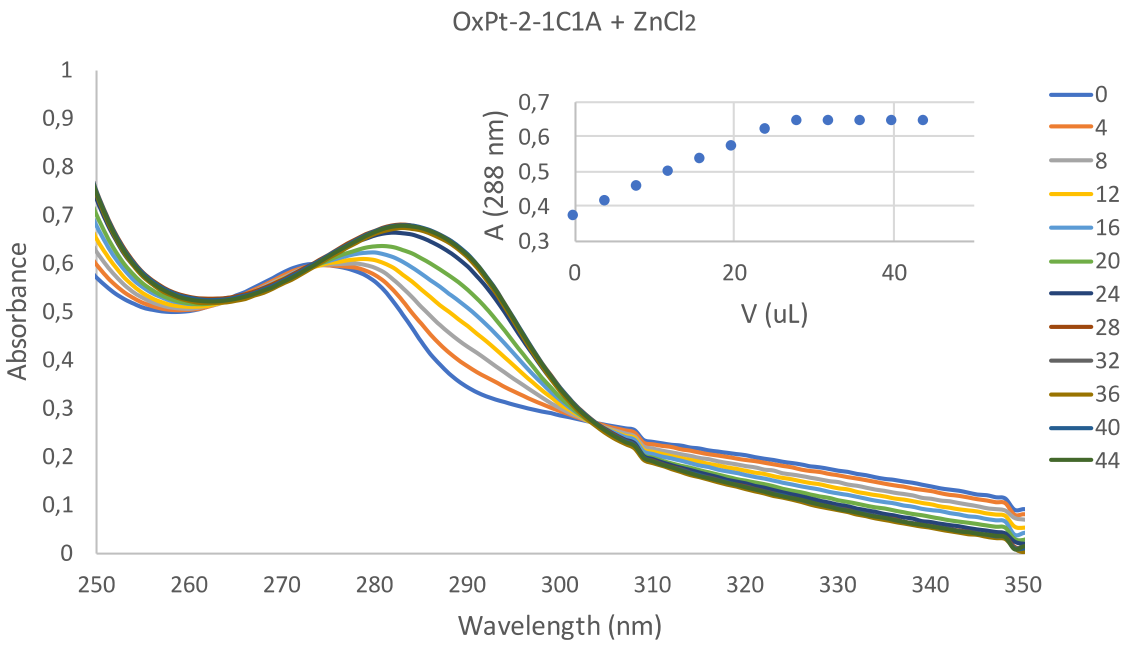

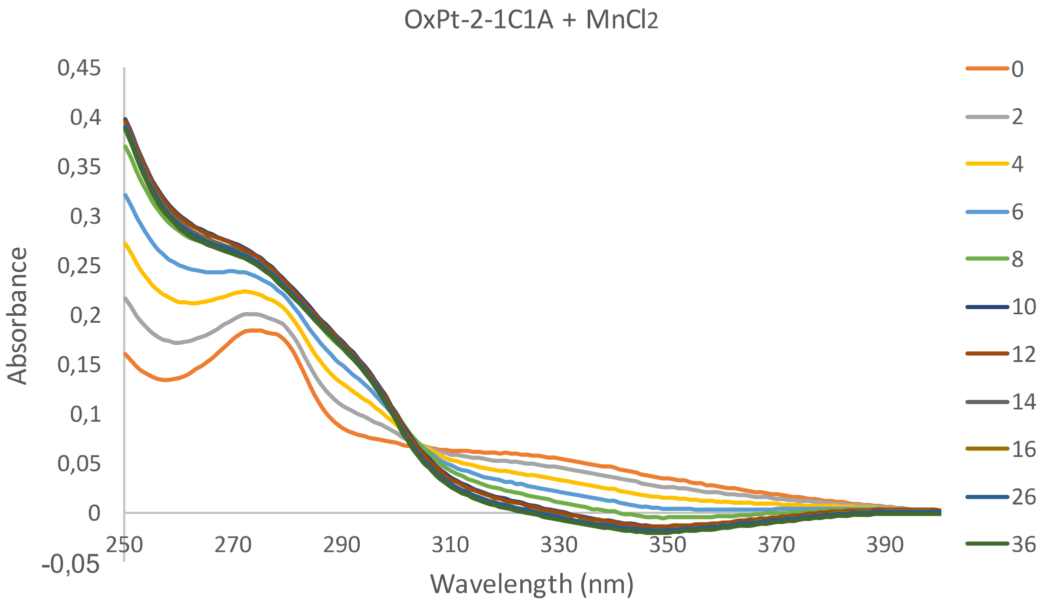

<u>Figure S4.</u> Titration curves of OxPt-2-1C1A. Top: with $ZnCl_2$ (volume added in µL, 10 mM in HEPES 50 mM pH 7.4) with plot of the absorbance of 288 nm as a function of $ZnCl_2$ volume added as insert; Bottom: with $MnCl_2$ (volume added in µL, 10 mM in HEPES 50 mM pH 7.4).

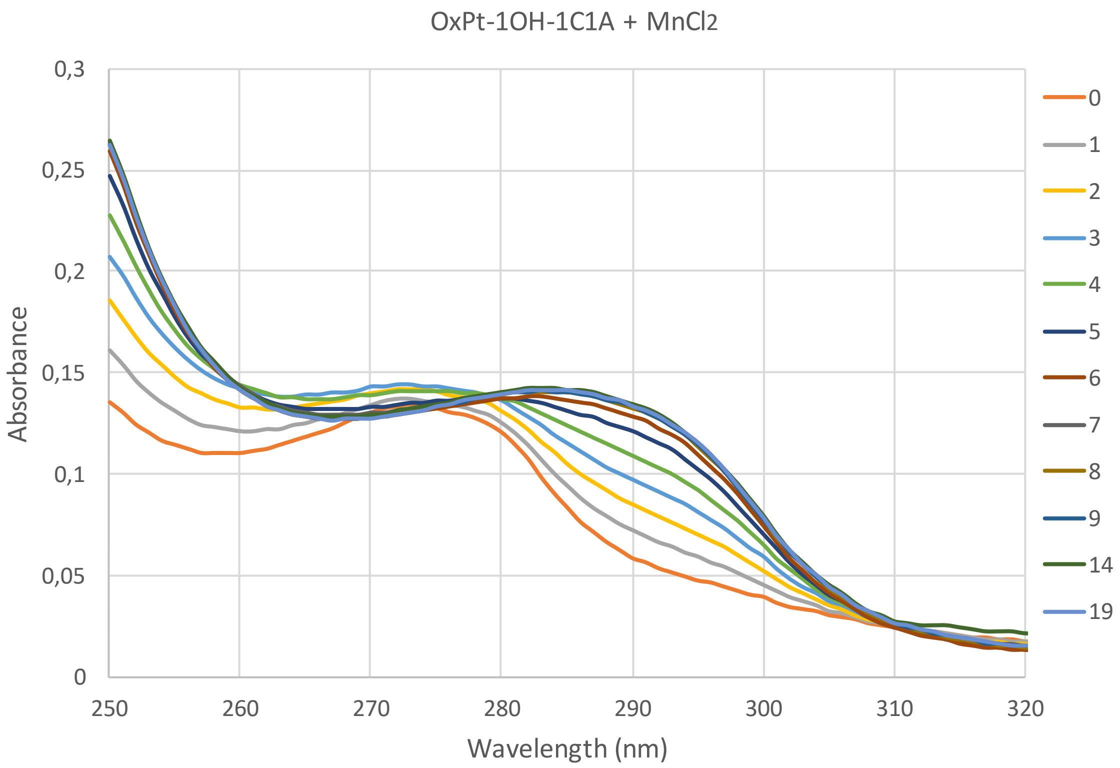

Figure S5. Titration curves of OxPt-1OH-1C1A with $MnCl_2$ (volume added in µL, 10 mM in HEPES 50 mM pH 7.4).

- Stability followed by HPLC:

The Figures below show the HPLC chromatograms for OxPt-1-1C1A and OxPt-2-1C1A as examples of signal evolution with time in different conditions (presence of $MnCl_2$ (1 or 2 eq.), Na Ascorbate (excess) or both). Graph of the time evolution used to determine half-lives $t_{1/2}$ are then given.

OxPt-1-1C1A:

OxPt-1-1C1A (ref)

+Mn
t 0

2h

6h

20h

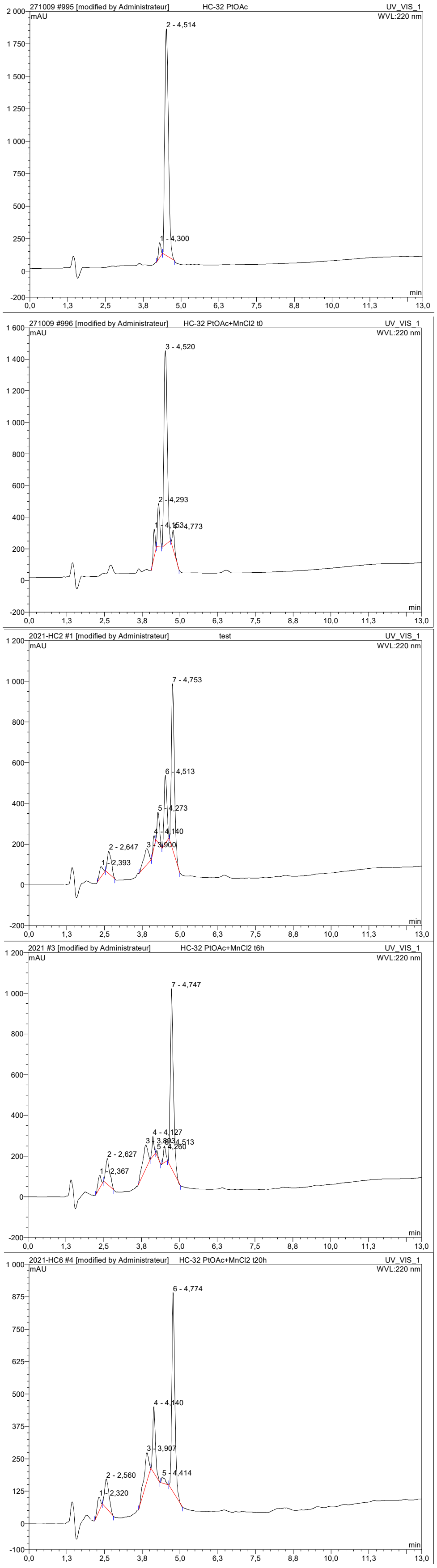

Figure S6. HPLC chromatograms (220 nm) of the conjugate OxPt-1-1C1A in the presence of $MnCl_2$ (1.0 eq) with time.

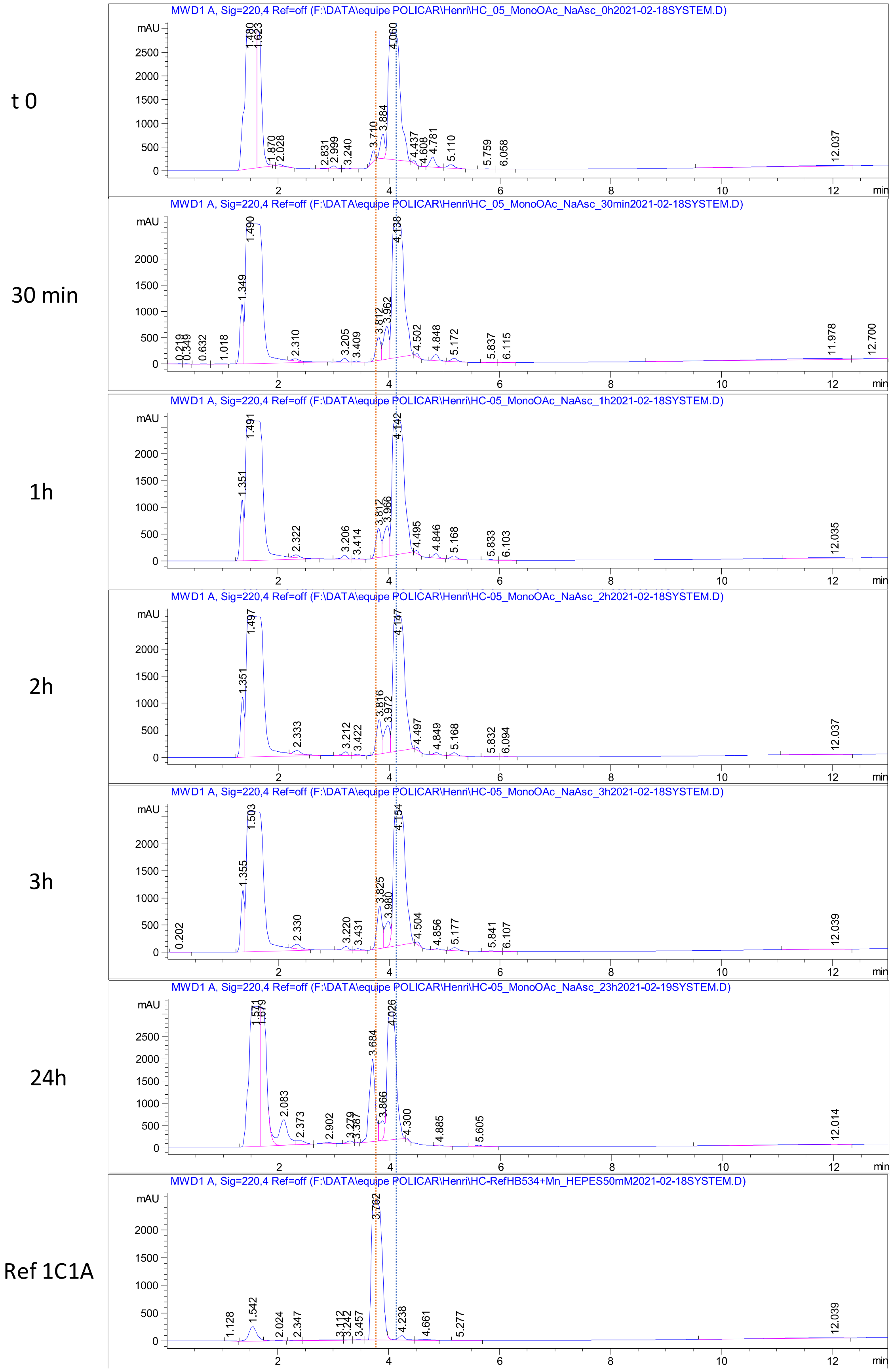

Figure S7. HPLC chromatograms (220 nm) of the conjugate OxPt-1-1C1A in the presence of NaAsc (30-fold excess) with time and HPLC signature of ligand 1C1A. Blue dotted line points the signal of OxPt-1-1C1A and orange dotted line the signal of ligand 1C1A.

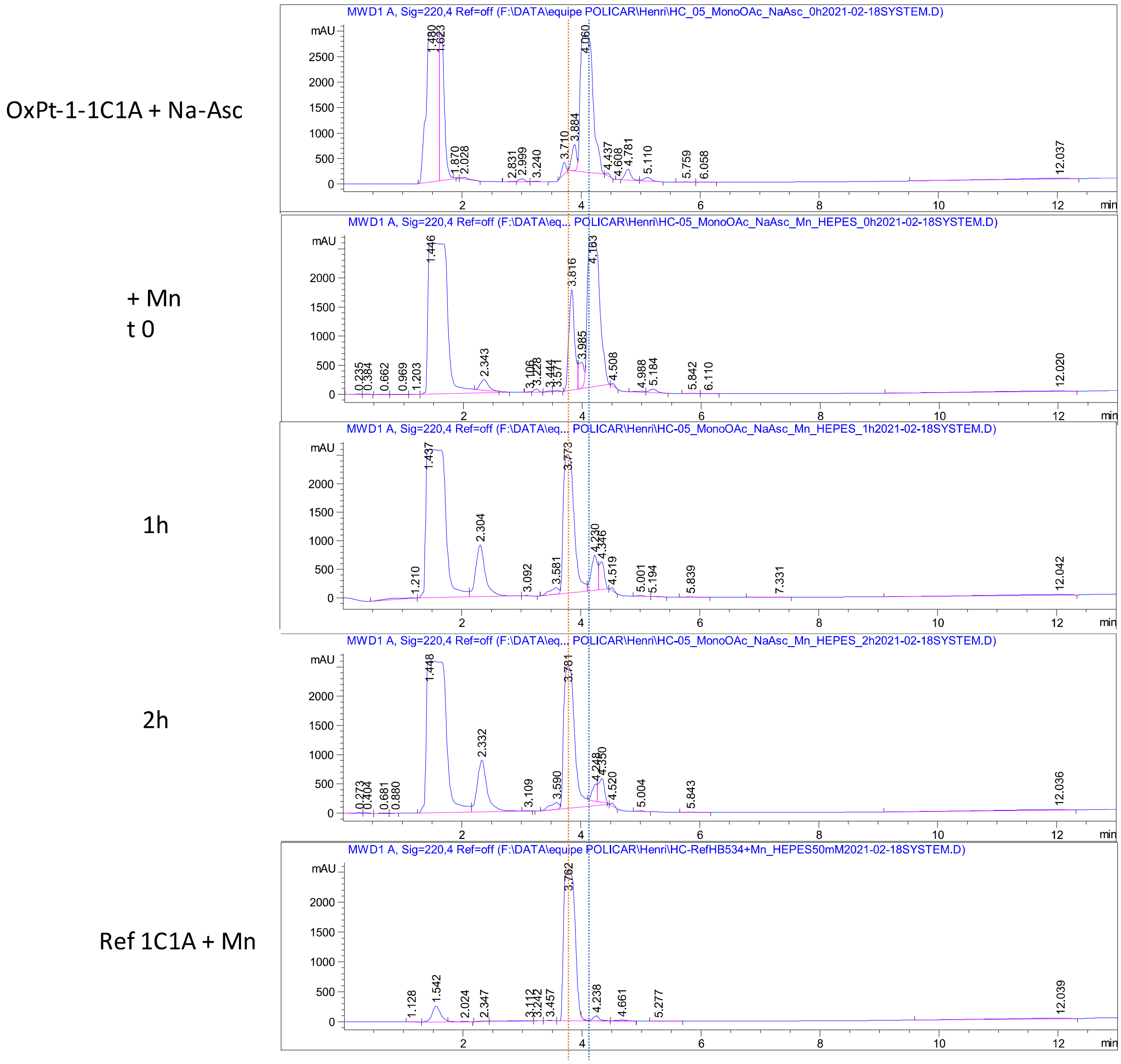

Figure S8. HPLC chromatograms (220 nm) of the conjugate OxPt-1-1C1A in the presence of NaAsc (30-fold excess) and $MnCl_2$ (1.0 eq) with time and HPLC signature of the complex Mn1C1A as reference. Blue dotted line points the signal of OxPt-1-1C1A and orange dotted line the signal of ligand 1C1A.

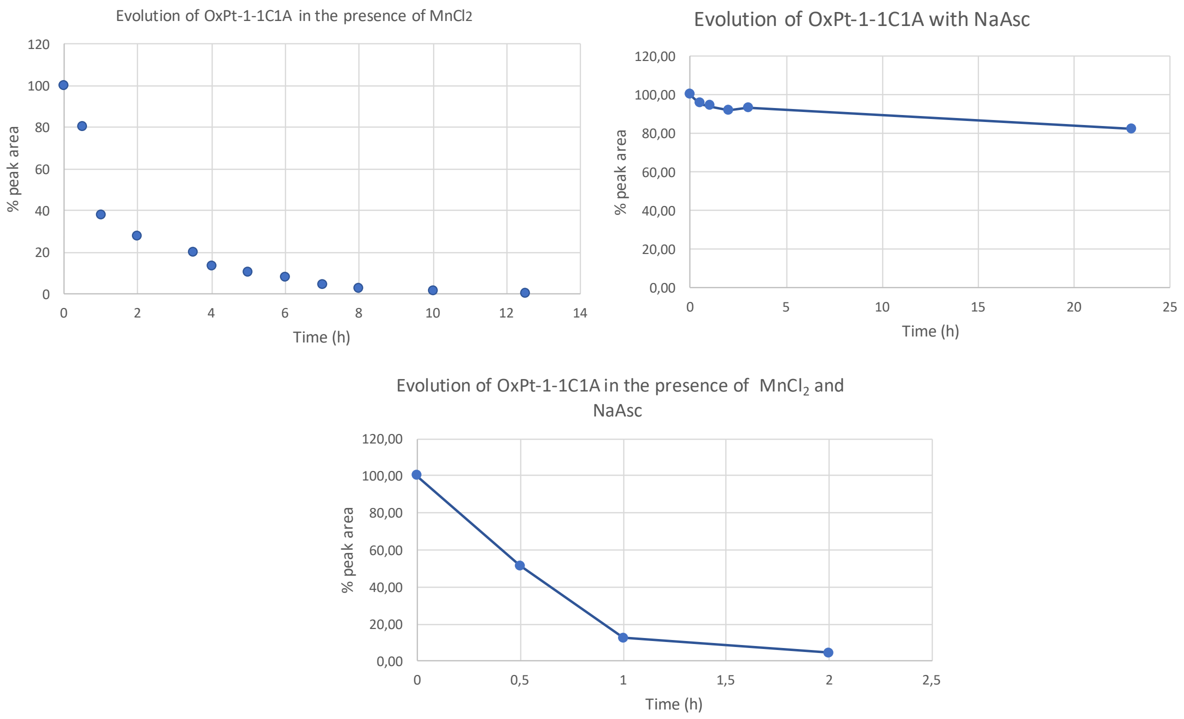

Figure S9. Evolution of the HPLC signal of the conjugate OxPt-1-1C1A at 220 nm with time.

OxPt-2-1C1A:

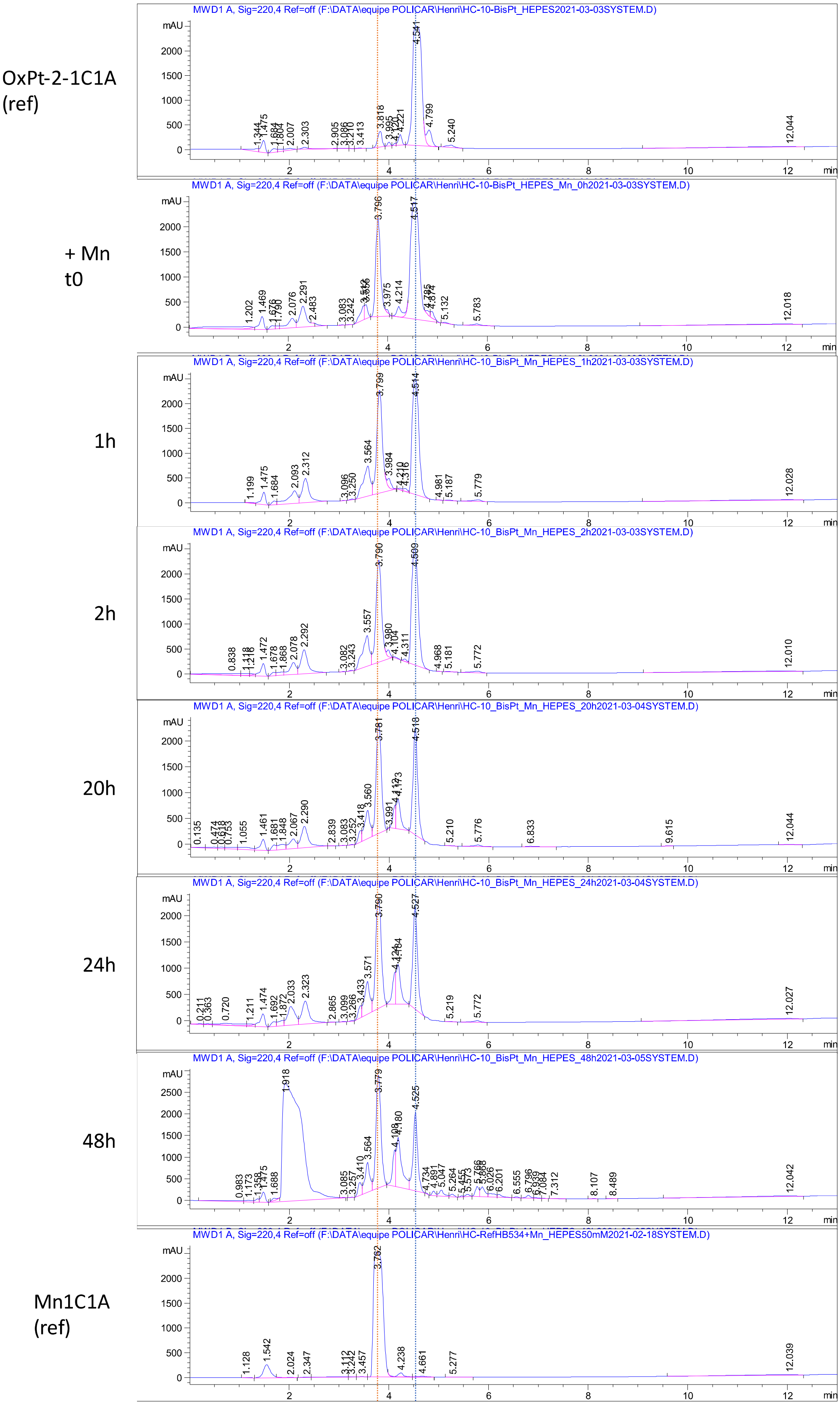

Figure S10. HPLC chromatograms (220 nm) of the conjugate OxPt-2-1C1A in the presence of $MnCl_2$ (2.0 eq) with time. Blue dotted line points the signal of OxPt-2-1C1A and orange dotted line the signal of the complex Mn1C1A.

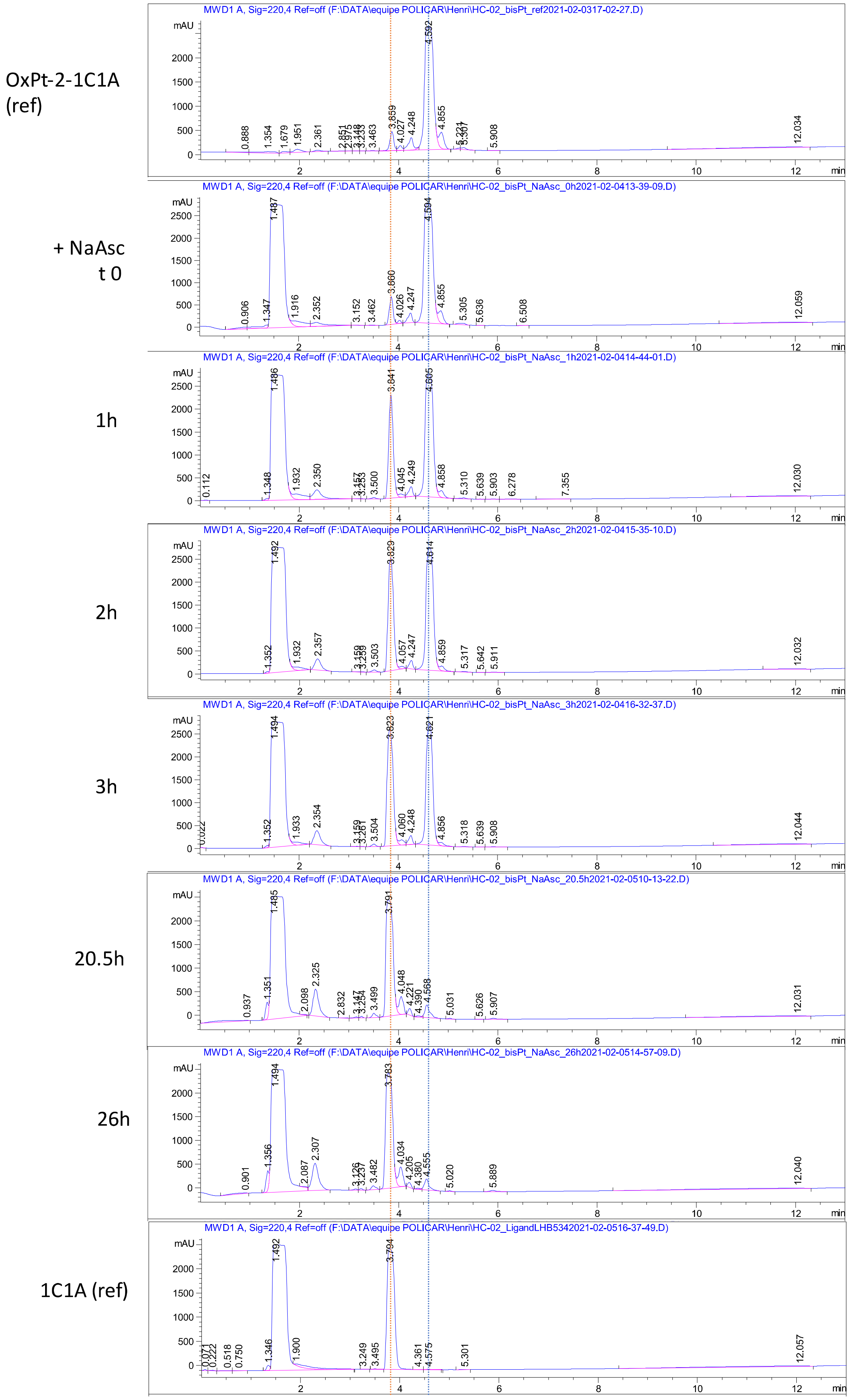

Figure S11. HPLC chromatograms (220 nm) of the conjugate OxPt-2-1C1A in the presence of NaAsc (30-fold excess) with time and HPLC signature of the ligand 1C1A as reference. Blue dotted line points the signal of OxPt-2-1C1A and orange dotted line the signal of ligand 1C1A.

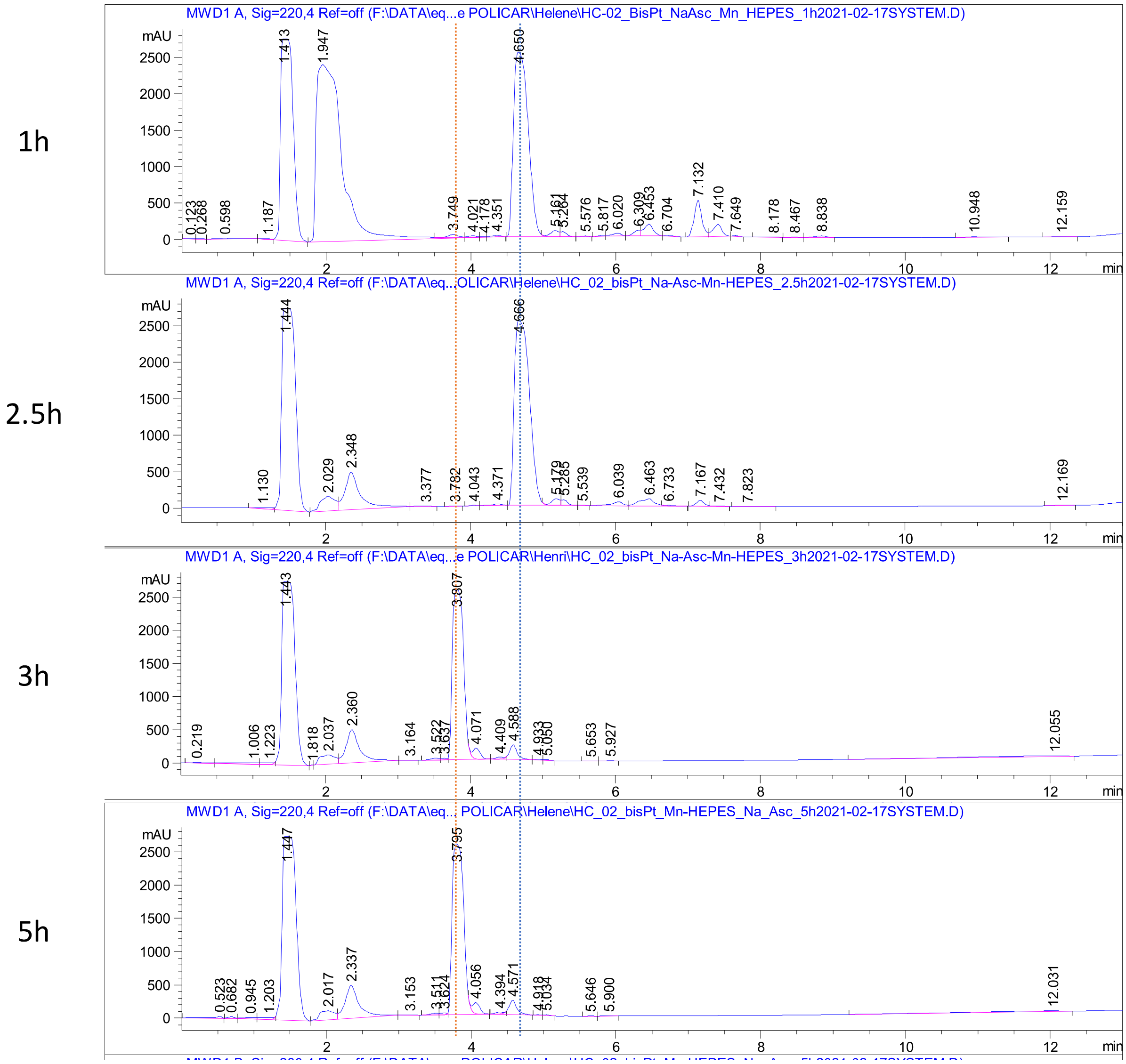

Figure S12. HPLC chromatograms (220 nm) of the conjugate OxPt-2-1C1A in the presence of NaAsc (30-fold excess) and $MnCl_2$ (1.0 eq) with time. Blue dotted line points the signal of OxPt-2-1C1A and orange dotted line the signal of ligand 1C1A.

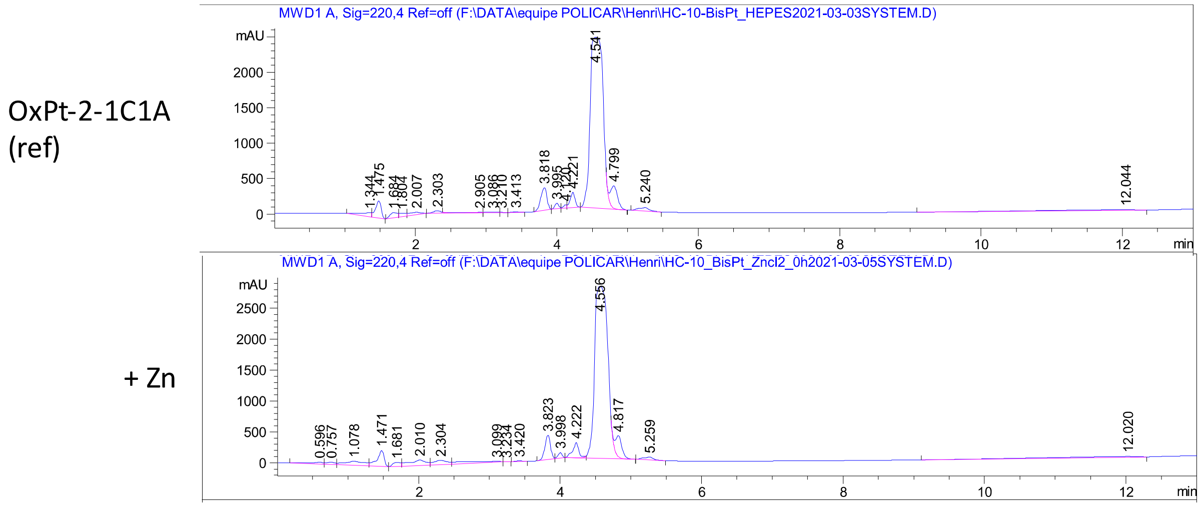

A

OxPt-2-1C1A

+ Zn

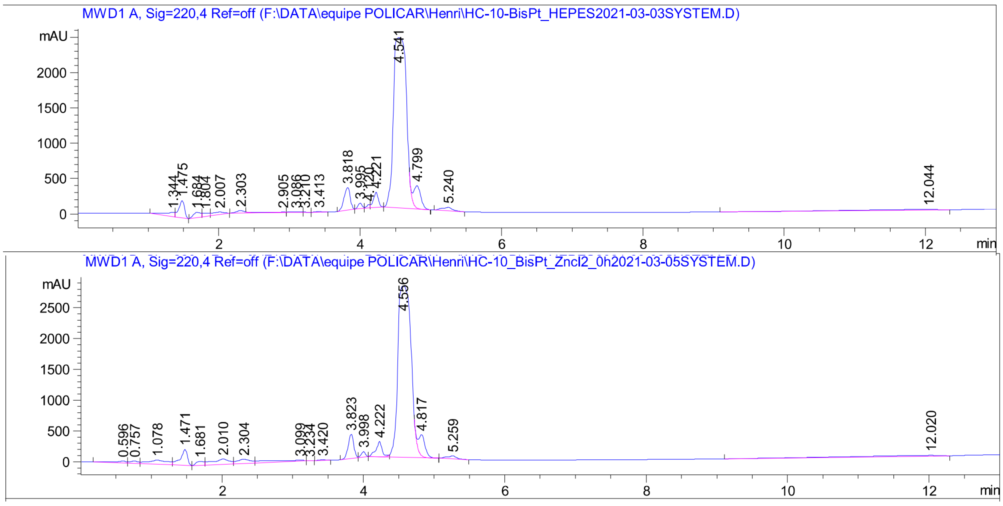

B

Pt(dach)(ox)(OH)$_2$

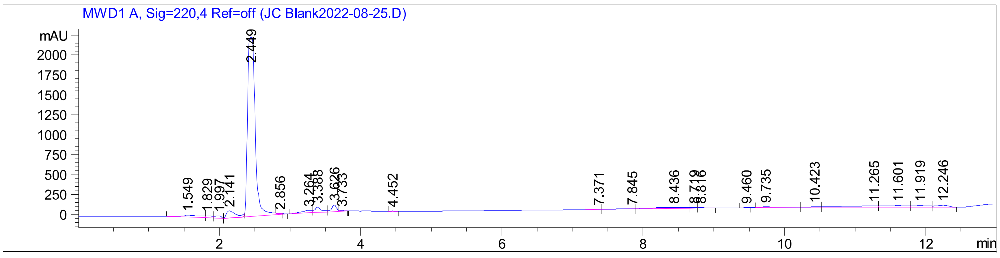

Oxaliplatin

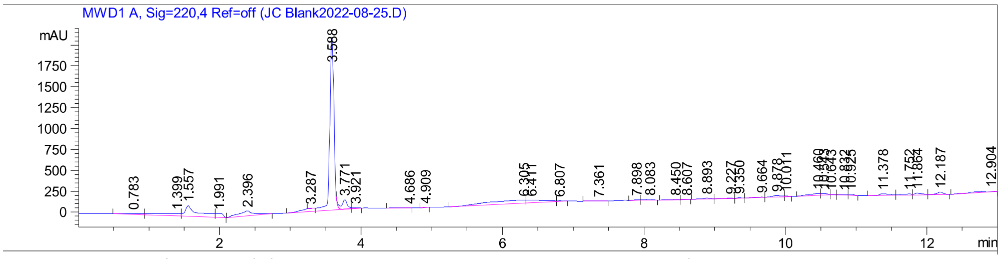

Figure S13. HPLC chromatograms (220 nm) (C18 column, 5 to 100% ACN in 10 min) of **A.** The conjugate OxPt-2-1C1A in the presence of $ZnCl_2$ (2.0 eq). No degradation occurs; B. Pt^{IV}(dach(ox)(OH)$_2$ and oxaliplatin.

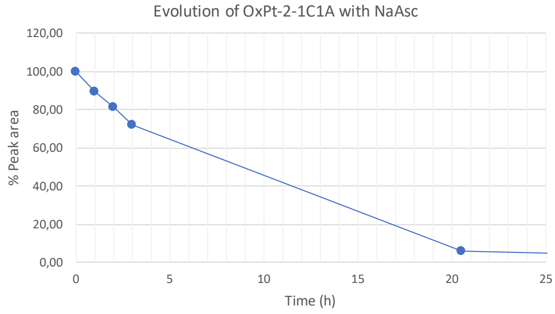

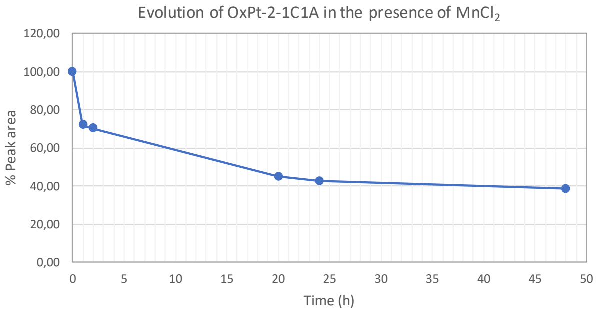

Figure S14. Evolution of the HPLC signal of the conjugate OxPt-2-1C1A at 220 nm with time in different conditions.

OxPt-1OH-1C1A :

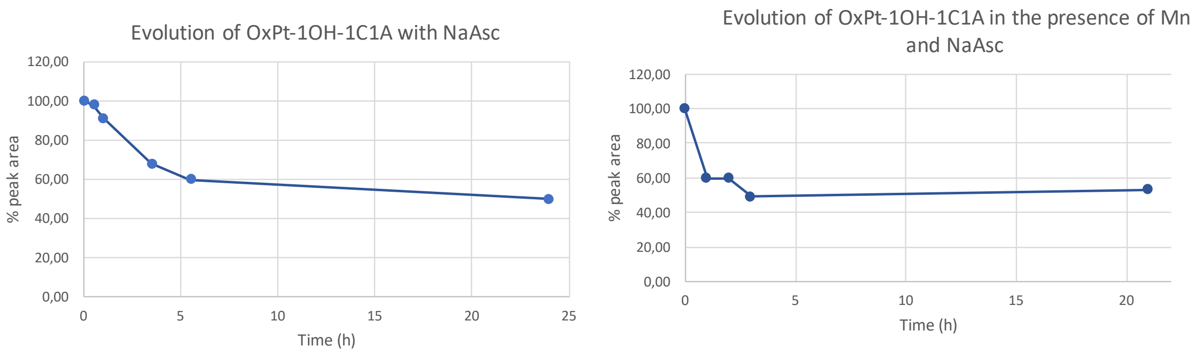

Figure S15. Evolution of the HPLC signal of the conjugate OxPt-1OH-1C1A at 220 nm with time in different conditions.

- Mass spectrometry

Mass spectroscopy analyses of ligand 1C1A and conjugate OxPt-1-1C1A were performed with and without $MnCl_2$ and the fragments detected were compared. The 100% peaks observed for ligand 1C1A are as expected the mono-protonated ligand when injected alone, the Mn or the Zn complex when pre-incubated with $MnCl_2$ or $ZnCl_2$ respectively (Figures S16-S18).

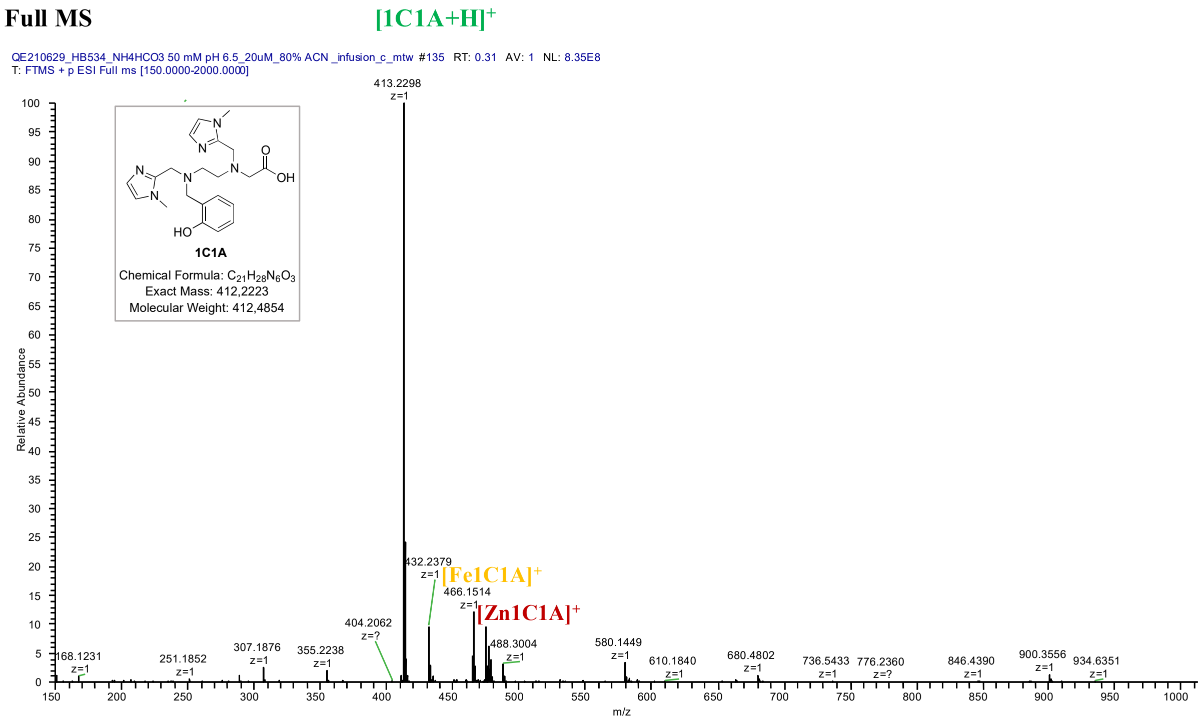

Figure S16. MS spectrum of **1C1A** (20 µM, 80% ACN / 20% NH_4HCO_3 50 mM pH 6.5).

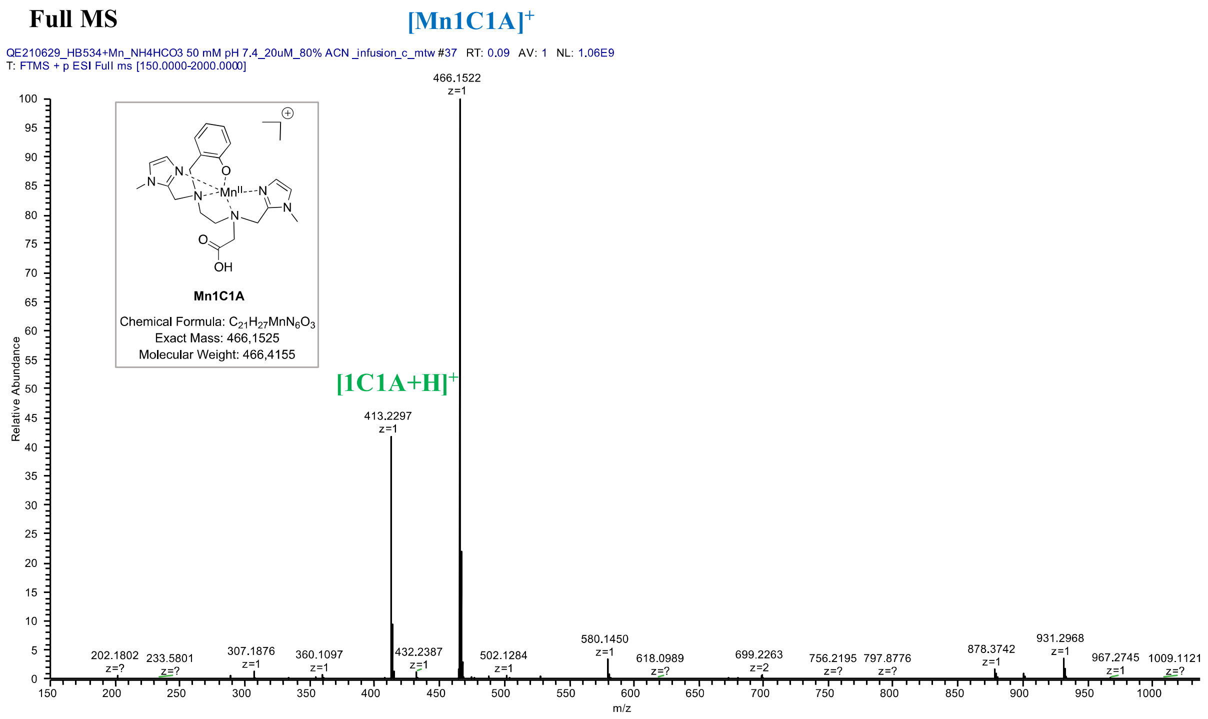

Figure S17. MS spectrum of **Mn1C1A** (20 µM, 80% ACN / 20% NH_4HCO_3 50 mM pH 7.4).

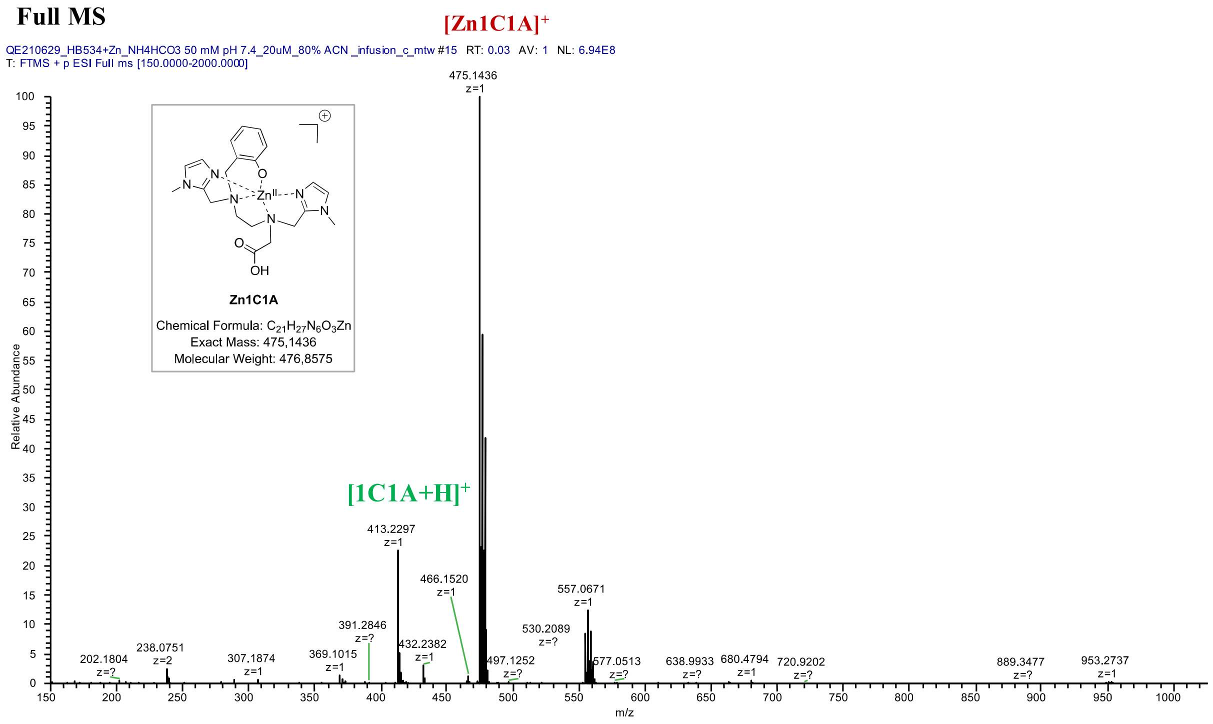

Figure S18. MS spectrum of **Zn1C1A** (20 µM, 80% ACN / 20% NH_4HCO_3 50 mM pH 7.4).

For OxPt-1-1C1A without Mn, the 100 % peak was the bis-protonated (doubly charged) ion derived from OxPt-1-1C1A (Figure S19). When mixed with Mn for 3 days (corresponding to full degradation time according to HPLC), the main detected signal was that of the Mn complex of ligand 1C1A (Mn1C1A) as a mono charged species, thus with a Mn at the +3 redox state (Figure S20). These data concur with a degradation of the conjugates in the presence of Mn and are consistent with the UV-Vis titration experiments.

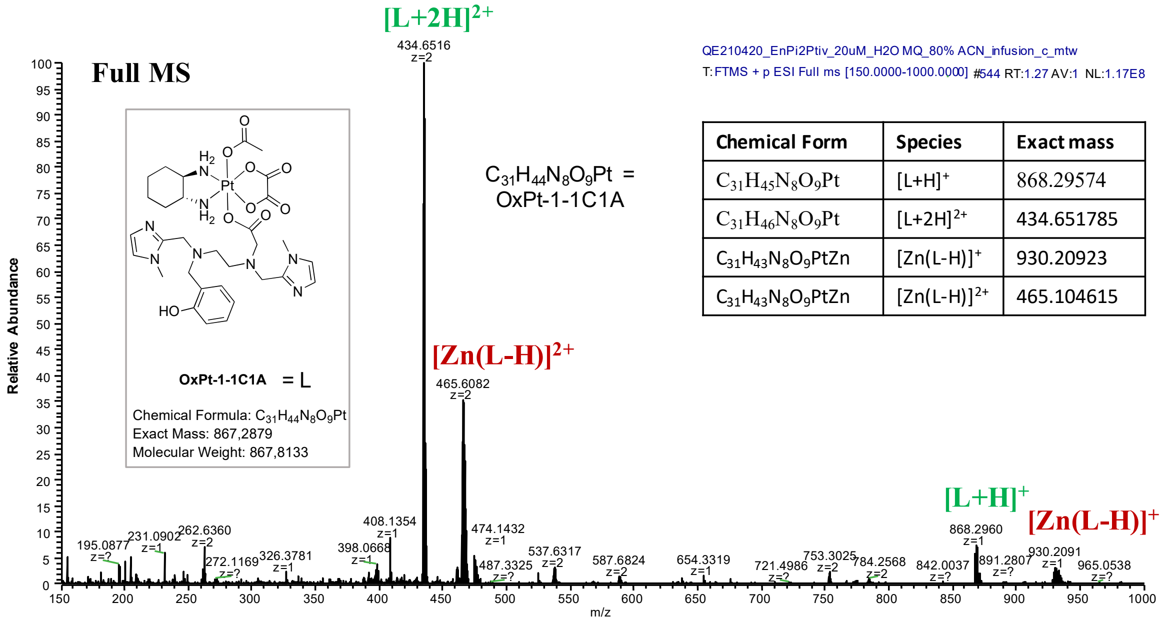

Figure S19. MS spectrum of **OxPt-1-1C1A** (20 µM, 80% ACN / 20% H_2O MQ).

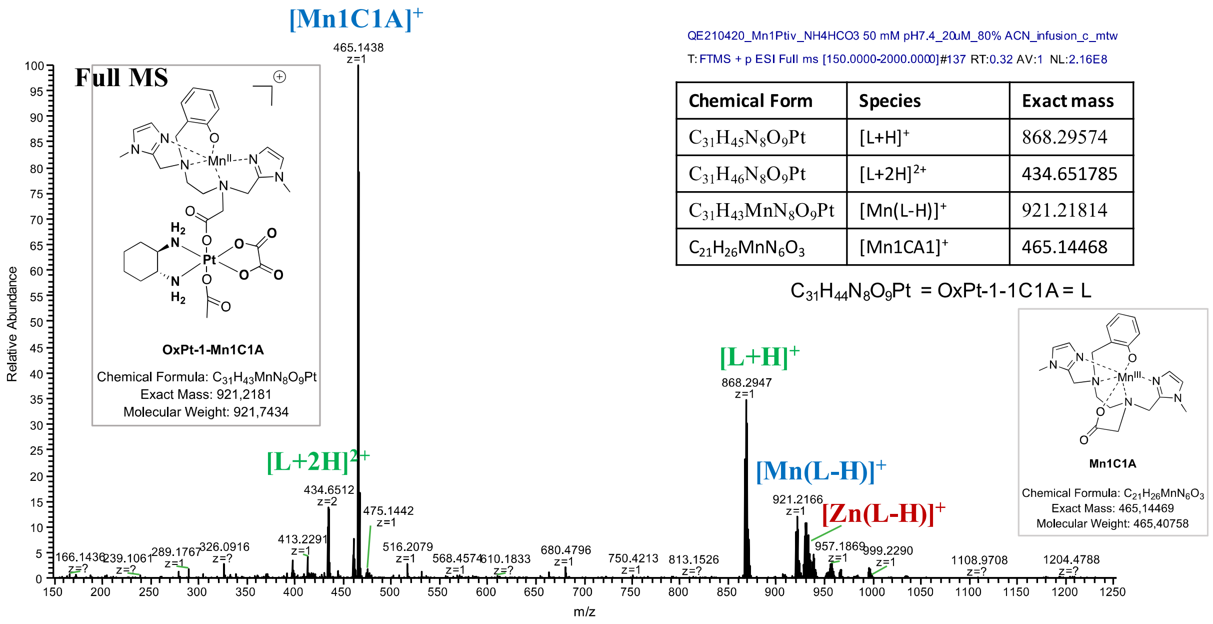

Figure S20. MS spectrum of **OxPt-1-Mn1C1A** after 3 days (80% ACN / 20% NH_4HCO_3 50 mM pH 7.4).

We propose the driving force of the reactivity with Mn to originate from the proximity between the ester link and the Mn ion in the SOD mimic ligand, prompting hydrolysis of the Pt-O bond to form a favourable 5-membered metallacycle between the carboxylate function of ligand **1C1A** and Mn. Although we failed in our attempts to crystallize ligand **1C1A** in the presence of Mn, we hypothesize the structure of the complex formed as a six-coordinated Mn^{III} complex as in Figure S20.

In the presence of Zn in the same conditions as above, the Zn^{2+} complex of OxPt-1-1C1A was detected as main fragment, in accordance with the stability observed in HPLC and the UV-Vis titration experiments (Figure S21). This can be explained by the redox inactive character of

Zn^{2+}, the intermediate character of Zn^{2+} in HSAB theory (versus hard metal for Mn^{2+}) and thus a weaker preference for carboxylate hard ligand and a preferred coordination number of 5.

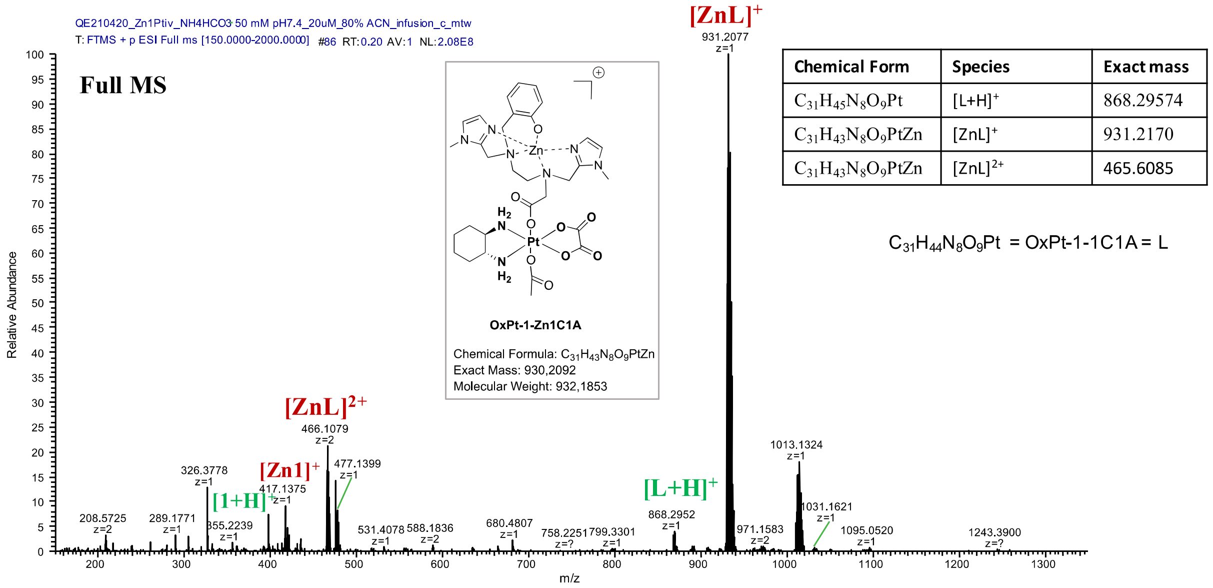

Chemical Form	Species	Exact mass
$C_{31}H_{45}N_8O_9Pt$	$[L+H]^+$	868.29574
$C_{31}H_{43}N_8O_9PtZn$	$[ZnL]^+$	931.2170
$C_{31}H_{43}N_8O_9PtZn$	$[ZnL]^{2+}$	465.6085

$C_{31}H_{44}N_8O_9Pt$ = OxPt-1-1C1A = L

Figure S21. MS spectrum of **OxPt-1-Zn1C1A** after 3 days (80% ACN / 20% NH_4HCO_3 50 mM pH 7.4).

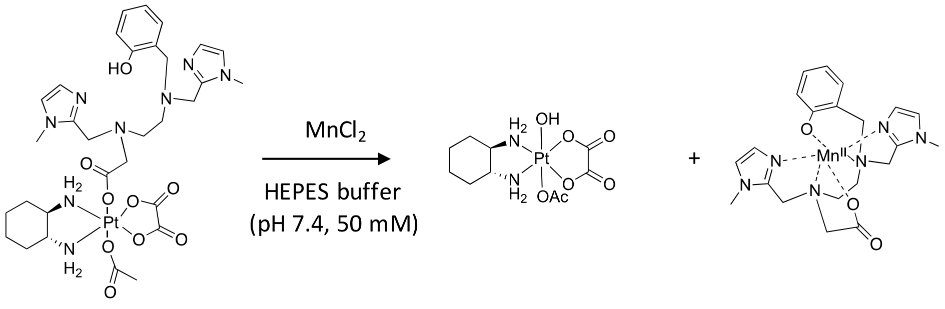

OxPt-1-1C1A

Pt(IV) species (and further hydrolysis)

Mn1C1A

Figure S22. Proposed reactivity of the Pt(IV) conjugate OxPt-1-1C1A with $MnCl_2$ as an example.

- Superoxide measurements in cells

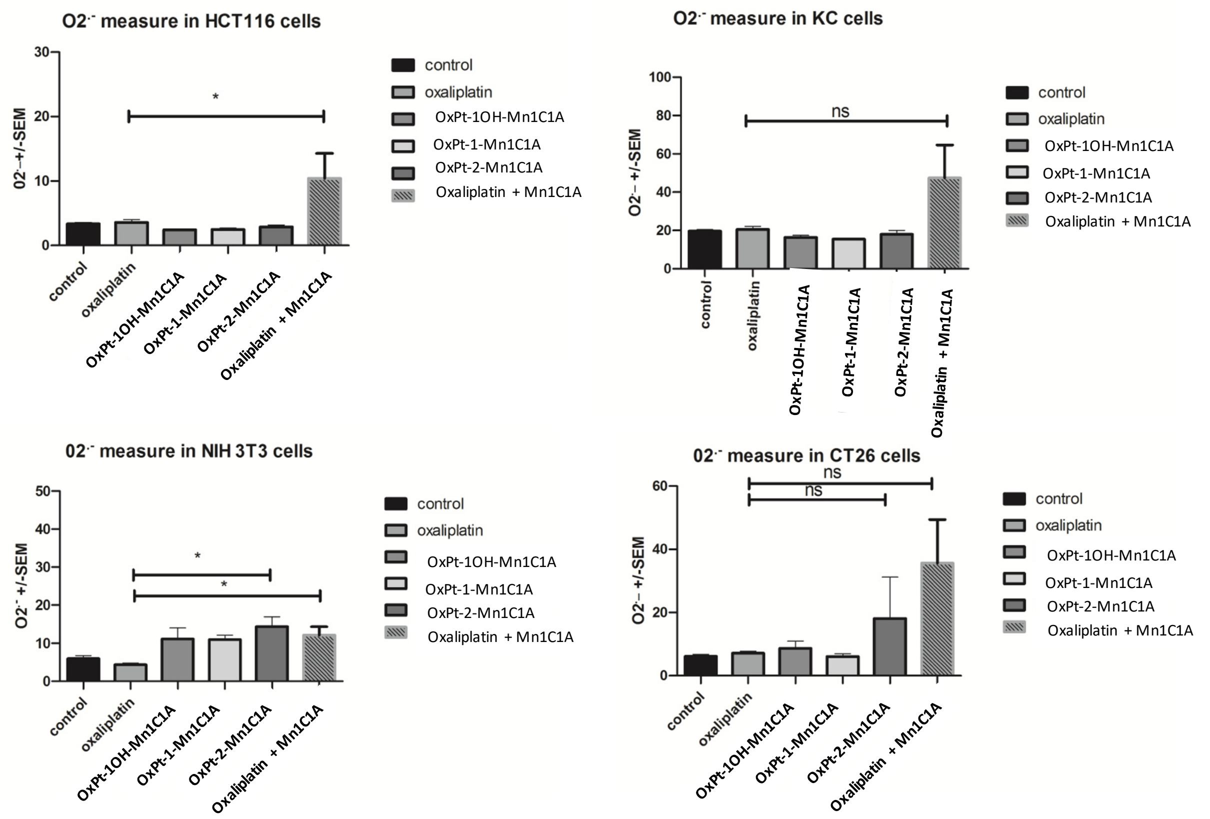

Figure S23. Mean $O_2^{\cdot -}$ levels *in vitro*. Mean $O_2^{\cdot -}$ levels measured by spectrometry (dihydroethidium (DHE)) in cell lines treated with vehicle, oxaliplatin, OxPt-1OH-Mn1C1A, OxPt-1-Mn1C1A, OxPt-2-Mn1C1A, Mn1C1A + oxaliplatin at 2.5 µM, 5 µM and 10 µM for 24 hours. The p values are denoted as follows: *p<0.05, **p<0.01, ***p<0.001, ****p<0.0001.

In HCT 116 cells, $O_2^{\cdot -}$ levels were significantly higher in the Mn1C1A + oxaliplatin group than in all the other groups (p<0.05). In the NIH 3T3 cell cultures, $O_2^{\cdot -}$ levels were significantly higher in the Mn1C1A + oxaliplatin group and in the OxPt-2-Mn1C1A group than in cells treated with oxaliplatin alone (p<0.05). No significative difference was observed in KC and CT26 cells.

- GSH metabolism in cells

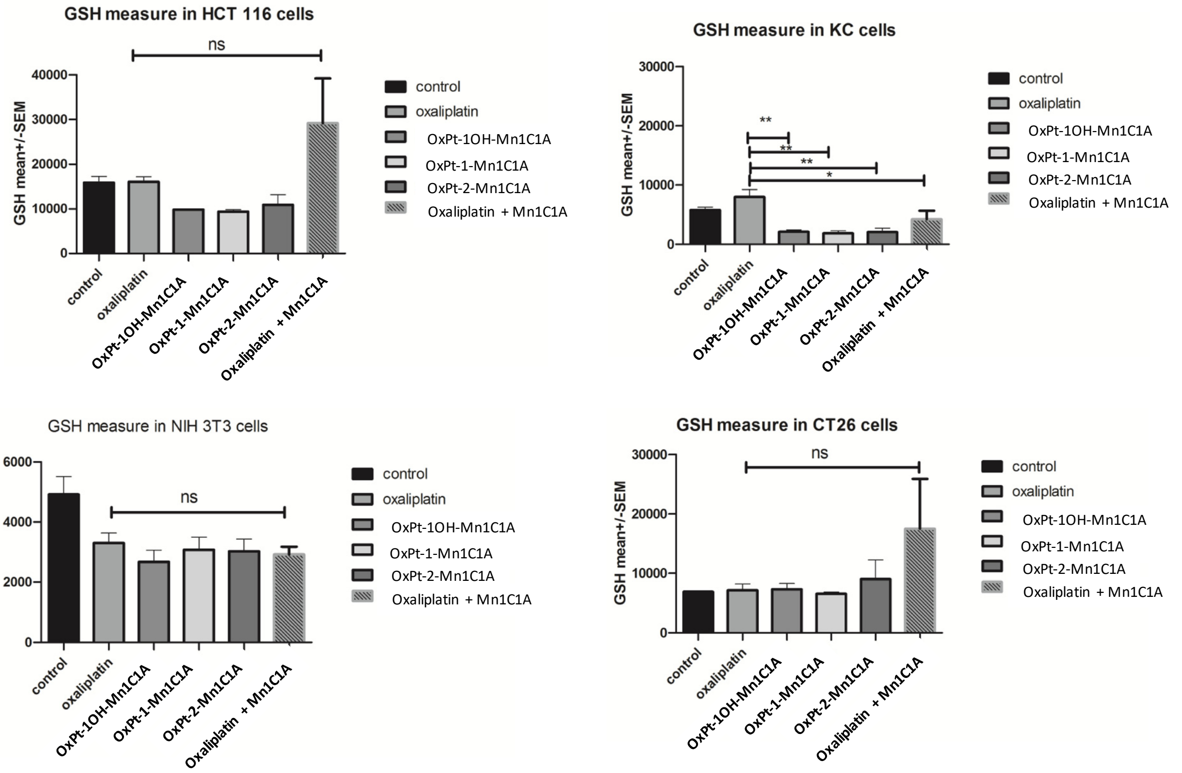

Figure S24. Mean GSH levels. Mean GSH levels measured by spectrometry (monochlorobimane) in cell lines treated with vehicle, oxaliplatin, OxPt-1OH-Mn1C1A, OxPt-1-Mn1C1A, OxPt-2-Mn1C1A, Mn1C1A + oxaliplatin at 2.5 µM, 5 µM and 10 µM for 24 hours. The p values are denoted as follows: *p<0.05, **p<0.01, ***p<0.001, ****p<0.0001.
